# Artificially Sweetened Beverage Consumption and Cancer Risk: A Comprehensive Dose–Response Meta-Analysis of Prospective Studies

**DOI:** 10.3390/nu14214445

**Published:** 2022-10-22

**Authors:** Tongxin Yin, Jiaoyuan Li, Yi Wang, Ke Liu, Tingting Long, Liming Cheng

**Affiliations:** Department of Laboratory Medicine, Tongji Hospital, Tongji Medical College, Huazhong University of Science and Technology, Wuhan 430030, China

**Keywords:** artificially sweetened beverage, low-calorie sweetener, sugar-sweetened beverage, cancer, risk

## Abstract

The impact of artificially sweetened beverages (ASBs), alternatives to sugar-sweetened beverages, on cancer incidence remains controversial. We conducted a meta-analysis of prospective studies to assess the association of daily ASB intake with cancer risk. A systematic search was performed between January 1967 and September 2022. Risk ratios (RR) or hazard ratios (HR) were extracted and pooled. The Grading of Recommendations, Assessment, Development, and Evaluations (GRADE) approach was used for the assessment of the certainty of evidence. The study was registered at PROSPERO (CRD42022312171). Overall, 14 articles with 17 cohorts were included. There was no significant association between daily ASB consumption and risk of overall cancer (highest versus lowest category: *n* = 17, *RR* = 1.03, 95% CI: 0.96–1.11, *p* = 0.407). For site-specific cancer analysis, the risk of non-lymphoid leukemia was elevated with high ASB intake (*n* = 3, *RR* = 1.35, 95% CI: 1.03–1.77, *p* = 0.030), while risk of colorectal cancer was decreased (*n* = 3, *RR* = 0.78, 95% CI: 0.62–0.99, *p* = 0.037). Dose–response analysis indicated a positive linear association between ASB intake and the risk of leukemia (*p_-linear_* = 0.027). The risk increased by 15% per one serving (355 mL) daily ASB intake increment (*RR* = 1.15, 95% CI: 1.02–1.30). In conclusion, ASB consumption might be positively associated with the risk of leukemia and negatively associated with the risk of colorectal cancer.

## 1. Introduction

High sugar-sweetened beverage (SSB) consumption was one of the risk factors for the overall attributable burden of death between 1990 and 2017 [1]. Collectively, SSBs contribute to the largest amount of added sugar in the diet [2]. Some SSBs are reported to have high Glycemic Index (GI) values, which produce rapid increases in blood glucose and insulin concentrations [3,4]. Studies have demonstrated the positive association of a high GI diet with the risk of chronic diseases [5,6,7,8]. Growing evidence suggests that SSB intake can induce hypertension [9], obesity [10], and type 2 diabetes [11] and can be associated with both the incidence and mortality of cancer, such as the risk of breast cancer, hepatocellular carcinoma, colorectal cancer, and prostate cancer and the mortality of breast cancer [12,13]. Owing to the potential health hazard of sugar, artificially sweetened beverages (ASBs), which are also referred to as low-calorie beverages or diet drinks, have become more and more popular in recent years [14]. Instead of simple sugar, ASBs use low-calorie sweeteners (LCS) such as aspartame, sucralose, or some polyols to induce perceptions of sweetness [15,16]. Compared with caloric sugar added in SSBs, the same amount of LCSs can trigger 200 times more sweetness than sucrose [16]. Therefore, LCSs are potent stimulators of sweetness, which means ASBs consume a much lower level of LCS for the equivalent sweetening power of SSBs, thus providing a negligible amount of energy. In addition, some LCSs, such as polyols, are not fully absorbed from the gut and thus are less available for energy metabolism [17] and do not cause a sudden increase in blood glucose compared with simple sugars. Hence, ASBs are popular among people with obesity for reducing caloric intake and people with diabetes for controlling blood sugar levels.

Though initially considered a healthier alternative to SSBs, the long-term health implications of ASBs remain to be answered [18]. Some LCSs were addressed to induce glucose intolerance by altering the gut microbiota [19]. Metabolic changes were also suggested, as LCS exposure might activate sweet taste receptors in oral and extraoral tissues, including intestine and pancreatic beta-cells [15], which could also stimulate insulin secretion [16]. A meta-analysis reported a positive association between ASB intake and the risk of diabetes, obesity, hypertension, and all-cause mortality [20]. As for cancer, in vitro studies have suggested the potential carcinogenic role of LCSs through inducing angiogenesis or DNA strand breaks [21,22,23]. In animal models, LCSs, including aspartame and sucralose, were demonstrated to have the capacity to induce hematopoietic tumors [24,25]. However, other in vitro studies have failed to show the carcinogenic activity of aspartame [26,27,28]. The inconsistent results raise questions regarding the potential role of ASBs and LCSs in carcinogenesis.

In accordance with in vitro and in vivo studies, results from epidemiological studies were unable to reach a consensus. A case–control study suggested that patients with bladder cancer had more frequent ASB consumption than healthy controls [29] Coincidentally, a significant association was found between heavy LCS use and higher-grade, poorly differentiated bladder cancer [30]. Nevertheless, several subsequent case–control studies found negligible associations of ASBs with the risk of bladder and other cancers [31,32,33,34]. In cohort studies, inconsistent results were also shown about the risk of hematopoietic cancers [35,36,37]. A recent cohort study observed an increased risk of obesity-related cancers with high ASB consumption [38], while another study did not support the involvement of ASBs in obesity-related cancers [39]. A meta-analysis published in 2021 reported a null association between ASB intake and overall cancer risk in subgroup analysis [40]. However, that meta-analysis used data from both prospective and retrospective studies, which increased the possibility of recall or selection bias. Meanwhile, several cohort studies [38,41,42,43,44] were published recently. Furthermore, the potential dose–response relationship between ASB intake and cancer risk has not been explored before. Additionally, a systematic evaluation of the quality assessment is also required. Hence, we conducted a meta-analysis of cohort studies to estimate the association as well as the dose–response relationship of ASB consumption with the risk of both overall and site-specific cancers and made a comprehensive evaluation of the certainty of evidence to strengthen the credibility of our results.

## 2. Materials and Methods

This work was conducted according to the Preferred Reporting Items for Systematic Reviews and Meta-Analyses (PRISMA) guidelines [45], and the review has been preregistered in PROSPERO (CRD42022312171).

### 2.1. Search Strategy

The literature search for articles published between January 1967 and September 2022 was conducted using the PubMed and Web of Science databases by two independent investigators. The following search terms were used without language or calendar date restriction: (“artificially sweetened beverage” OR “beverage, artificially sweetened” OR “sweetened beverage, artificially” OR “diet drink” OR “drink, diet” OR “diet beverage” OR “beverage, diet” OR “low caloric soft drink” OR “noncaloric soft drink” OR “low caloric soft soda” OR “noncaloric soft soda” OR “diet soft soda” OR “non-nutritive sweetener” OR “artificial sweetener” OR “low caloric sweetener” OR “cyclamate” OR “aspartame” OR “saccharin” OR “neotame” OR “alitame” OR “sucralose” OR “acesulfame-K”) AND (“cancer” OR “tumor” OR “carcinoma” OR “neoplasm” OR “malignancy”) AND (“prospective” OR “cohort” OR “longitudinal” OR “follow-up”). The reference lists of reviews and eligible studies were checked as well for additional studies. 

### 2.2. Study Selection

Studies were included if they met the following criteria: (1) studies with a prospective cohort design; (2) ASB intake as the exposure; (3) cancer incidence as the outcome; and (4) reported estimates of risk ratio (RR) or hazard ratio (HR) and corresponding 95% confidence intervals (CIs) in the highest versus lowest categories. Studies that treated the exposure as a continuous variable were excluded, as *RR*s or *HR*s for the highest versus lowest category could not be obtained. For dose–response analysis, studies should provide a quantitative measurement of intake, the number of cases, and follow-up person-years for each category (or sufficient data to calculate them) with at least three categories classified based on the dosage of exposure. Two authors performed the study selection independently, and disagreements were resolved by the intervention of a third author.

### 2.3. Data Extraction

The following data were extracted from each study by two independent authors: first author, year of publication, cancer type, age and sex at entry, cohort name, area, follow-up duration, number of cases, number of participants, exposure assessment, exposure dosages, minimally and maximally adjusted *RR*s or *HR*s and corresponding 95% CIs for all categories, and confounding variables adjusted in multivariable analysis. For the highest versus lowest category comparison, we extracted information about the classification of the highest and lowest categories and the levels of daily ASB consumption in these two groups, respectively. The mean or median daily intake of ASBs, number of cases, and follow-up person-years in each intake category were further extracted for dose–response analysis. The method described by D. Aune et al. was used to estimate person-years for each category in studies that only reported the total number of person-years and distribution of cases [46]. The most updated study was used for analysis when the same study participants reported outcomes more than once. For beverage consumption, the mean or median intake level was assigned to the corresponding *RR* for each category. If only ranges of intake were reported, the average of the lower and upper boundaries was calculated as an estimated midpoint. When the highest category was open-ended, the average intake level was assigned at 1.5 times the lower boundary. When the lowest category was open-ended, the average intake level was estimated by dividing the higher boundary by 1.5 [47]. ASB intake was converted from different units into milliliters per day (mL/d) based on the study-specific serving size. When ASB intake was reported as g/d, we considered 12 oz (355 mL) equivalent to 336 g [48]. When the serving size was reported by can or glass, we referred to the area of each study and considered a 330 mL serving equivalent to one can or glass for European countries [49], 355 mL for the United States [43], and 375 mL for Australia [40]. When LCS intake was reported, we referred to the category of LCSs and considered every 30 mg of acesulfame potassium, 167 mg of aspartame, or 50 mg of sucralose intake equivalent to one can of ASB consumption [50]. Cancers were classified based on the International Classification of Diseases, 10th revision (ICD-10), and the International Classification of Diseases for Oncology, Second and Third Edition (ICD-O-2 and ICD-O-3). Obesity-related cancers were identified according to the report of the International Agency for Research on Cancer (IARC) [51].

### 2.4. Risk of Bias Assessment and Quality of Evidence

We used the preliminary Risk of Bias in Non-randomized Studies of Exposure (ROBINS-E) tool to assess the methodological quality of included studies [52]. According to ROBINS-E, seven domains were examined, including bias due to confounding, selection of participants, classification of exposures, deviation from intended exposures, missing data, measurement of outcomes, and selection of the reported results. Each domain was assessed as at low, moderate, serious, or critical ROB. The overall bias was assigned according to the most severe item-level judgment. The criteria adopted for the ROB assessment are presented in Supplementary Table S1.

The Grading of Recommendations Assessment, Development, and Evaluation (GRADE) approach was used to assess the certainty of the evidence, which comprises the following items: risk of bias (the results of the ROB assessment), inconsistency (*I*^2^ > 50.0% or *p* < 0.10, or unexplained interstudy heterogeneity), indirectness (presence of factors that limit the generalizability of the results), imprecision (the 95% CI for effect estimates crosses no effect and fails to exclude important benefit or important harm), and publication bias [53]. Studies were assessed as very low, low, moderate, or high certainty of evidence. By default, all of the studies started with a high-quality rating. After the above five domain assessment, final ratings were concluded through reasonable upgrades and downgrades.

Two independent authors answered signaling questions, judged ROB for each study, and conducted the quality assessment. Disagreements were resolved by the intervention of a third author.

### 2.5. Data Analysis

A random-effect model was used to estimate summary *RR*s and 95% CIs for the highest versus lowest levels of ASB intake from different cohorts. Separate *RR* estimates for various cancer types and subtypes in a cohort were firstly pooled using a fixed-effect model to obtain a single *RR* before pooling with results from other cohorts included in the meta-analysis. Q test and *I*^2^ test were used to assess between-study heterogeneity. *I*^2^ described the proportion of total variation attributable to between-study heterogeneity as opposed to random error or chance, and a value of ≤25%, 25–50%, 50–75%, and >75% indicated no, low, moderate, and significant heterogeneity, respectively [54]. Subgroup analyses were conducted according to characteristics of study subjects (area, sample size, age, sex, body mass index (BMI) at recruitment, duration of follow-up), measurements of exposure (ASB measurement approach and frequency), outcome assessment (ascertainment of cancer), and data analysis (levels of ASB consumption in controls, classifications of ASB consumption, adjustment regarding SSB intake and adiposity measures) to investigate the associations in certain subjects and to explore the potential sources of heterogeneity.

Generalized least-squares trend estimation analyses were used for dose–response analysis, according to the methods developed by Orsini [55]. For nonlinear relationship assessment, we used restricted cubic splines with 4 knots at fixed percentiles (5%, 35%, 65%, and 95%) of the distribution [56].

Publication bias was estimated using Egger [57] and Begg [58] analyses and a visual inspection of the funnel plot. Sensitivity analysis was performed by excluding one study at a time sequentially to evaluate the influence of individual studies on the summary estimate.

All the *p*-values were two-sided, and a *p*-value of less than 0.05 was considered statistically significant. All statistical analyses were undertaken using STATA/MP, version 14.0 (Stata Corporation, College Station, TX, USA).

## 3. Results

### 3.1. Literature Search

**Figure 1:**
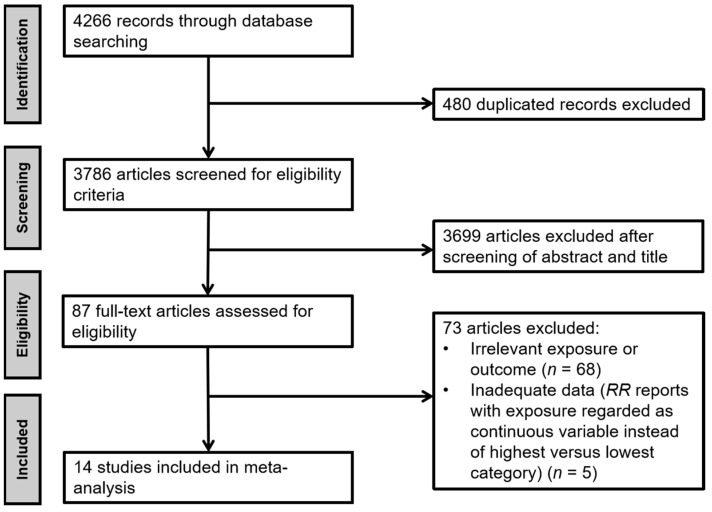
Flow chart of literature research and study selection.

The process of study identification and selection is presented in Figure 1. Overall, 4266 reports were identified by searching the PubMed and Web of Science databases, of which 480 were excluded as duplicates. Among these, 3699 articles were excluded through title or abstract screening, and 87 full-text articles of potentially relevant studies were identified. Sixty-eight articles were further excluded due to irrelevant exposures or outcomes and five due to inadequate data (*RR* reports with exposure regarded as a continuous variable instead of highest versus lowest category), resulting in 14 eligible articles. No additional studies were identified after checking the reference lists of included studies.

### 3.2. Study Characteristics and Quality Assessment

Table 1 provides a summary of the study characteristics. The 14 articles including 17 cohort studies comprised a total of 34,050 cancer cases occurring in 2,821,922 participants [35,36,37,38,39,42,43,44,48,59,60,61,62,63]. Seven cohorts reported the association between ASB intake and the risk of more than one type of cancer. Cases were diagnosed with breast cancer (*n* = 4), non-Hodgkin lymphoma (*n* = 4), pancreatic cancer (*n* = 4), colorectal cancer (*n* = 3), non-lymphoid leukemia (*n* = 3), multiple myeloma (*n* = 3), endometrial cancer (*n* = 2), prostate cancer (*n* = 2), gastric cancer (*n* = 1), gliomas (*n* = 1), Hodgkin lymphoma (*n* = 1), kidney cancer (*n* = 1), ovarian cancer (*n* = 1), and thyroid cancer (*n* = 1). All 17 cohorts reported maximally adjusted *RR*s or *HR*s, and 11 reported minimally adjusted *RR*s. The studies were published between 2006 and 2022, including 23,039 to 487,922 participants followed for 5.1 to 28.97 years. Ten of the cohorts reported separate *RR*s by sex. Eleven cohorts recruited participants from North America, four from Europe, and two from Australia. The assessment of ASB intake was performed using lifestyle questionnaires (*n* = 4) or validated Food Frequency Questionnaires (FFQs) (*n* = 13). Among the 17 included cohorts, 6 cohorts compared the lowest and highest quintiles, 5 compared the lowest and highest quartiles, 3 compared the lowest and highest sextiles, and the other 3 used the lowest tertile as a reference. Partly due to the inconsistent classification of category, ASB consumption in the lowest category of 9 cohorts was none, and the remaining 8 cohorts had low levels of ASB consumption in the lowest category group. *RR*s were adjusted for sociodemographic (i.e., age, sex, race, educational level, socioeconomic position) and lifestyle factors (i.e., dietary intake, physical activity, smoking status). Thirteen cohorts further adjusted for adiposity measures (i.e., weight, BMI, waist circumference). Three cohorts did not adjust for adiposity measures, and one did not adjust for lifestyle factors. More detailed information on the confounders for each study is presented in Table 1.

The grades for ROB in the seven domains and the detailed reasons for assessments are exhibited in Supplementary Table S2, and the study-level ROB is summarized in Supplementary Table S3. For the overall study-level ROB judgment, all 14 articles were assigned as moderate ROB. The most common reason for bias was the lack of direct measurement of exposure, with most information obtained through questionnaires.

### 3.3. ASB Consumption and Risk of Overall Cancer

A total of 17 cohorts reported an association between ASB intake and the risk of different types of cancers. We found a null association of high ASB intake with maximal adjusted risk of overall cancer (*n* = 17, highest versus lowest category, pooled *RR* = 1.03, 95% CI: 0.96–1.11, *p* = 0.407, *I*^2^ = 53.0%). Fifteen cohorts that reported three or more categories of adequate ASB intake values were enrolled for dose–response analysis. There was no significant linear (*n* = 15, *p_-linear_* = 0.316, Supplementary Figure S1A) or nonlinear (*p_-nonlinear_* = 0.515) relationship between ASB intake and overall cancer risk.

Minimally adjusted associations between ASB consumption and the risk of overall and site-specific cancers are presented in Supplementary Table S4. The studies produced a pooled *RR* of 1.07 for overall cancers (*n* = 11, highest versus lowest category, 95% CI: 0.97–1.20, *p* = 0.297, *I*^2^ = 70.6%).

### 3.4. ASB Consumption and Risk of Hematopoietic Cancers

**Figure 2:**
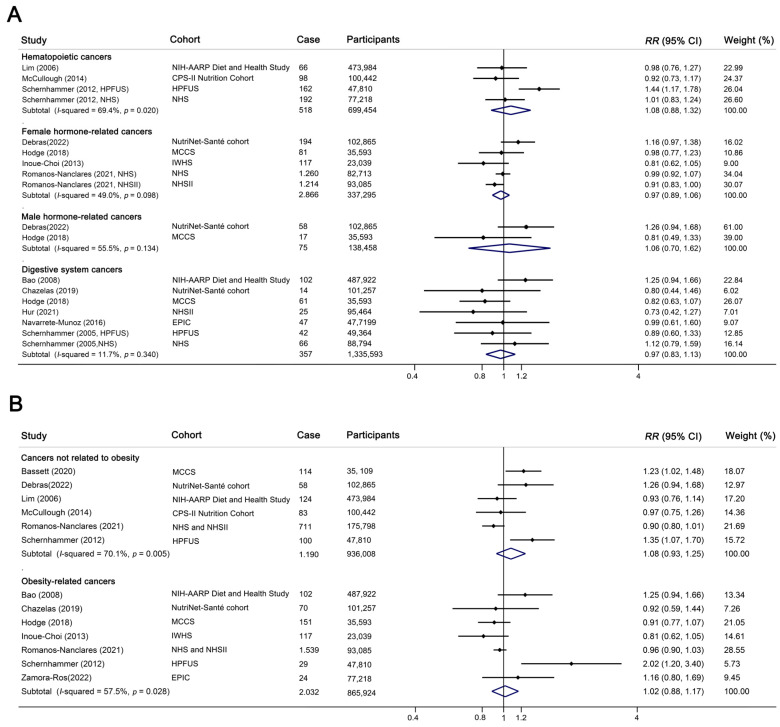
Forest plot of artificially sweetened beverage (ASB) intake and risk of cancer [35,36,37,38,39,42,43,44,48,59,60,61,62,63]. Dots indicate adjusted *RR*s by comparing the highest with the lowest categories of intake, and diamonds indicate the pooled *RR*s. The size of the shaded square is proportional to the percentage weight of each study, and horizontal lines indicate 95% CIs. Overall *RR*s calculated with random-effect model. Cases of cancer in lowest versus highest categories and participants for each study are presented. (**A**) Forest plot of ASB intake and risk of hematopoietic cancers (*n* = 4), female hormone-related cancers (*n* = 5), male hormone-related cancers (*n* = 2), and digestive system cancers (*n* = 7). (**B**) Forest plot of ASB intake and risk of obesity-related cancers (*n* = 7) and non-obesity-related cancers (*n* = 6). Abbreviation: NIH-AARP Diet and Health Study, the National Institutes of Health–AARP Diet and Health Study; CPS-II Nutrition Cohort, the Cancer Prevention Study-II Nutrition Cohort; HPFUS, the Health Professionals Follow-Up Study; NHS, the Nurses’ Health Study; MCCS, the Melbourne Collaborative Cohort Study; IWHS, the Iowa Women’s Health Study; NHS II, the Nurses’ Health Study II; EPIC, and European Prospective Investigation into Cancer and Nutrition.

Four cohorts reported association of ASB consumption with the risk of hematopoietic cancers, including non-lymphoid leukemia (*n* = 3), multiple myeloma (*n* = 3), non-Hodgkin (*n* = 3) and Hodgkin lymphoma (*n* = 1). All four cohorts were included to facilitate the dose–response analysis. Non-Hodgkin lymphoma contained common subtypes, which included chronic lymphocytic leukemia, small lymphocytic lymphoma, follicular lymphoma, and diffuse large B-cell lymphoma [35,36,37]. The studies produced a pooled *RR* of 1.08 (highest versus lowest category: 95% CI: 0.88–1.32, *p* = 0.480, Figure 2A, Supplementary Figure S2), with moderate heterogeneity between studies (*I*^2^ = 69.4%). There was no evidence of a linear (*p_-linear_* = 0.280, Supplementary Figure S1B) or nonlinear dose–response association (*p_-nonlinear_* = 0.309) between ASB consumption and the risk of overall hematopoietic cancer.

**Figure 3:**
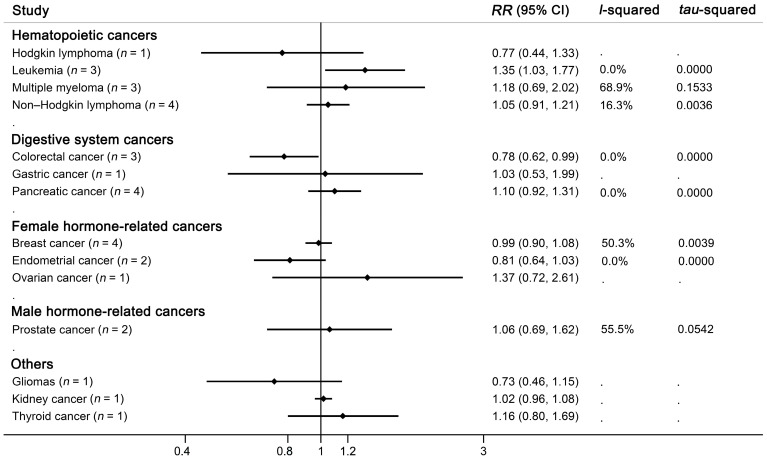
Forest plot of artificially sweetened beverage (ASB) intake and risk of site-specific cancers. Dots indicate the pooled adjusted *RR*s by comparing the highest with the lowest categories of intake. Pooled *RR*s were calculated with random-effect model.

**Figure 4:**
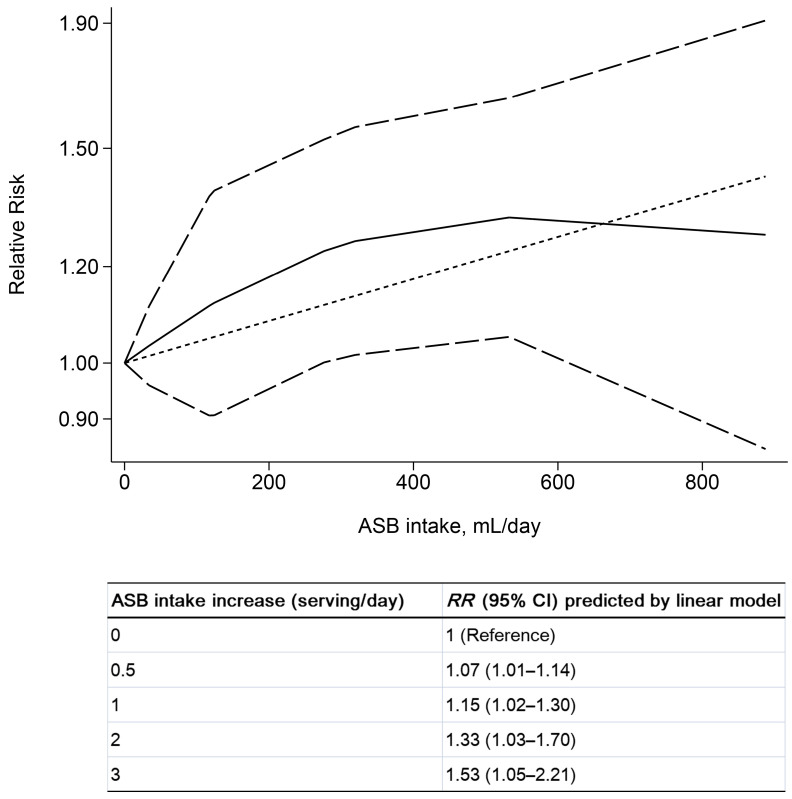
Dose–response analysis of artificially sweetened beverage (ASB) intake with risk of leukemia. The shot dash dot line represents linear modeled *RR*. The solid line and the long dash line represent the *RR* estimates and 95% CI of nonlinear model, respectively. Estimated *RR*s and 95% CIs for per 0.5, 1, 2, 3 servings (355 mL) daily ASB intake increments by linear model is presented in the table above.

A subgroup analysis showed a significant association of dietary ASB intake with leukemia (highest versus lowest category, *RR* = 1.35, 95% CI: 1.03–1.77, *p* = 0.030), with no indication for heterogeneity (*I*^2^ = 0.0%, Figure 3, Supplementary Figure S2). For dose–response analysis, we revealed a significant positive linear relationship between dietary ASB intake and leukemia (*p_-linear_* = 0.027, Figure 4). The risk for leukemia increased by 15% per one serving (355 mL) daily ASB intake increment (*RR* = 1.15, 95% CI: 1.02–1.30). No association was found between ASB intake and multiple myeloma (highest versus lowest category, *RR* = 1.18, 95% CI: 0.69–2.02, *p* = 0.537, *I*^2^ = 68.9%), non-Hodgkin lymphoma (highest versus lowest category, *RR* = 1.05, 95% CI: 0.91–1.21, *p* = 0.506, *I*^2^ = 16.3%), or Hodgkin lymphoma (highest versus lowest category, *RR* = 0.77, 95% CI: 0.45–1.33, *p* = 0.351, *I*^2^ = 0.0%).

### 3.5. ASB Consumption and Risk of Digestive System Cancers

Seven cohorts reported a risk of digestive system cancers, including cancer of the colorectum (*n* = 3), pancreas (*n* = 4), and gastric cardia (*n* = 1). The studies produced a pooled *RR* of 0.97 (*n* = 7, highest versus lowest category, 95% CI: 0.83–1.13, *p* = 0.672, *I*^2^ = 11.7%, Figure 2A, Supplementary Figure S2). All seven cohorts were enrolled for dose–response analysis, and the results suggested that there was no significant linear (*p_-linear_* = 0.930, Supplementary Figure S1C) or nonlinear (*p_-nonlinear_* = 0.438) relationship between ASB intake and the risk of cancers in the digestive system.

Risk of colorectal cancer decreased with high ASB intake with low heterogeneity between studies (highest versus lowest category, *RR* = 0.78, 95% CI: 0.62–0.99, *p* = 0.037, *I*^2^ = 0.0%, Figure 3, Supplementary Figure S2), with no evidence of linear (*p_-linear_* = 0.117) or nonlinear (*p_-nonlinear_* = 0.055) relationship. No significant association was found in cancer of gastric cardia (highest versus lowest category, *RR* = 1.03, 95% CI: 0.53–1.99, *p* = 0.930) or pancreas (highest versus lowest category, *RR* = 1.10, 95% CI: 0.92–1.31, *p* = 0.307, *I*^2^ = 0.0%).

### 3.6. ASB Consumption and Risk of Hormone-Related Cancers

Five cohorts reported the association between ASB intake and hormone-related cancer, including cancer of the breast (*n* = 4, including both premenopausal and postmenopausal breast cancer), endometrium (*n* = 2), ovary (*n* = 1), and prostate (*n* = 2). For female hormone-related cancer, the studies produced a pooled *RR* of 0.97 (*n* = 5, highest versus lowest category, 95% CI: 0.89–1.06, *p* = 0.509, *I*^2^ = 49.0%, Figure 2A). For prostate cancer, the studies produced a pooled *RR* of 1.06 (*n* = 2, highest versus lowest category, 95% CI: 0.70–1.62, *p* = 0.785, *I*^2^ = 55.5%). Site-specific cancer analysis showed no significant association between ASB intake and the risk of cancer of the breast (highest versus lowest category, *RR* = 0.75, 95% CI: 0.9–1.08, *p* = 0.75, *I*^2^ = 50.3%), endometrium (highest versus lowest category, *RR* = 0.81, 95% CI: 0.64–1.03, *p* = 0.091, *I*^2^ = 0.0%), or ovary (highest versus lowest category, *RR* = 1.37, 95% CI: 0.72–2.61, *p* = 0.338).

The five cohorts that reported a risk of female hormone-related cancer were enrolled for dose–response analysis. The results suggested that there was no significant linear (*p_-linear_* = 0.124, Supplementary Figure S1D) or nonlinear (*p_-nonlinear_* = 0.150) relationship between ASB intake and female hormone-related cancer risk.

### 3.7. ASB Consumption and Risk of Other Cancers (Kidney Cancer, Thyroid Cancer, and Gliomas)

One cohort reported a non-significant association between ASB intake and kidney cancer, with an *RR* of 0.92 (*n* = 1, highest versus lowest category, 95% CI: 0.46–1.84, *p* = 0.814). One cohort demonstrated a null association between ASB intake and thyroid cancer, with an *RR* of 1.16 (*n* = 1, highest versus lowest category, 95% CI: 0.80–1.69, *p* = 0.437). The association between ASB consumption and risk of gliomas was also insignificant from one cohort study (*n* = 1, highest versus lowest category, *RR* = 0.73, 95% CI: 0.46–1.15, *p* = 0.178). 

### 3.8. ASB Consumption and Risk of Cancer Related to Obesity

The IARC currently identified thirteen cancers—i.e., esophagus (adenocarcinoma), gastric cardia, colon and rectum, liver, gallbladder, pancreas, breast (postmenopausal), corpus uteri, ovary, kidney (renal cell), meningioma, thyroid, and multiple myeloma—as linked to being overweight and obese with sufficient evidence [51]. To explore the association between ASB intake and the risk of cancer related to obesity, cohorts involving the cancers mentioned above were grouped into obesity-related cancers, and cohorts involving all other cancers were defined as non-obesity-related cancers in our study. No significant association was observed between ASB intake and the risk of obesity-related cancers (*n* = 9, highest versus lowest category, *RR* = 0.99, 95% CI: 0.87–1.12, *p* = 0.832, *I*^2^ = 48.7%) or non-obesity-related cancers (*n* = 6, highest versus lowest category, *RR* = 1.07, 95% CI: 0.93–1.25, *p* = 0.329, *I*^2^ = 70.1%, Figure 2B). Additionally, there was no evidence of a linear (*n* = 7, *p_-linear_* = 0.711 for obesity-related cancers, *n* = 6, *p_-linear_* = 0.339 for non-obesity-related cancers) or nonlinear (*p_-nonlinear_* = 0.708 for obesity-related cancers, Supplementary Figure S1E, *p_-nonlinear_* = 0.289 for non-obesity-related cancers, Supplementary Figure S1F) dose–response association.

### 3.9. Subgroup Analyses, Sensitivity Analyses, and Publication Bias

The results of subgroup analyses by characteristics of study subjects (area, sample size, age, sex, BMI at recruitment, duration of follow-up), measurements of exposure (ASB measurement approach and frequency), outcome assessment (ascertainment of cancer), and data analysis (levels of ASB consumption in controls, classifications of ASB consumption, adjustment regarding SSB intake, and adiposity measures) are shown in Table 2. The relationship between ASB intake and overall cancer risk was significant in Europeans (*n* = 4, highest versus lowest category, *RR* = 1.10, 95% CI: 1.01–1.19, *p* = 0.025, *I*^2^ = 0.0%) while not significant in Americans (*n* = 11, highest versus lowest category, *RR* = 1.00, 95% CI: 0.91–1.09, *p* = 0.936, *I*^2^ = 55.9%) and Australians (*n* = 2, highest versus lowest category, *RR* = 1.12, 95% CI: 0.92–1.38, *p* = 0.254, *I*^2^ = 44.7%). The risk of cancers increased in studies that used lifestyle questionnaires (*n* = 4, highest versus lowest category, *RR* = 1.10, 95% CI: 1.01–1.19, *p* = 0.025, *I*^2^ = 0.0%) instead of validated FFQs (*n* = 13, highest versus lowest category, *RR* = 1.02, 95% CI: 0.93–1.11, *p* = 0.708, *I*^2^ = 57.2%) for ASB intake measurement. The risk of cancers was also increased in studies with three-category classification of ASB consumption (*n* = 3, highest versus lowest category, *RR* = 1.12, 95% CI: 1.02–1.22, *p* = 0.018, *I*^2^ = 0.0%). No significant effect of ASB intake on cancer risk was observed in the other subgroups (all *p* > 0.05, Table 2). Sensitivity analysis suggested that the overall risk estimate was not substantially modified by any single study (Supplementary Figure S3), with the summary estimates ranging from 1.00 (95% CI: 0.95–1.07) to 1.05 (95% CI: 0.97–1.13).

No publication bias was observed between the 17 cohorts included in the meta-analysis on ASB intake and cancer risk through both the visual inspection of the funnel plot (Supplementary Figure S4) and the analyses suggested by Egger’s test (*p* = 0.783) and Begg’s test (*p* = 0.869).

### 3.10. Certainty of Evidence

A very low certainty of evidence supported the null association regarding ASB intake on the risk of overall cancer according to the GRADE table (Table 3). For site-specific cancer analysis, we found moderate certainty of evidence that higher ASB consumption was associated with an increased risk of leukemia, as the heterogeneity between studies was low (*I*^2^ = 0.0%), the 95% CI of the *RR* did not overlap the effect (highest versus lowest category, *RR* = 1.35, 95% CI: 1.03–1.77), and the positive dose–response relationship (*p_-linear_* = 0.027) upgraded the evidence by one level. A low certainty of evidence supported the negative association of ASB intake with the risk of colorectal cancer. The null associations between ASB intake and the risk of gliomas, multiple myeloma, and non-Hodgkin lymphoma showed low certainty of evidence. The remaining ten results displayed a very low certainty of the evidence, including the risk of Hodgkin lymphoma and cancer of the breast, endometrium, gastric cardia, kidney, ovary, pancreas, prostate, and thyroid.

## 4. Discussion

The current meta-analysis covered prospective cohort studies regarding ASB consumption and the risk of cancers and explored their potential dose–response relationships for the first time. Overall, we included 14 population-based prospective studies with a total of 2,821,922 participants. The results suggested that high ASB consumption increased the risk of leukemia with moderate certainty of evidence and decreased the risk of colorectal cancer with low certainty of evidence. For dose–response analysis, the risk for leukemia increased by 15% per one serving (355 mL) daily ASB intake increment.

Our results demonstrated a significant positive association between ASB consumption and increased risk of leukemia, which was consistent with a previous meta-analysis published in 2021 [40]. A series of studies from the Ramazzini Institute found rats fed with aspartame developed more lymphomas and leukemias [24,64,65,66]. Aspartame, especially in liquids near or above room temperature, can quickly break down into methanol and produce several kinds of noxious metabolites, including formaldehyde [67]. Formaldehyde has displayed mutagenic and genotoxic actions in several in vivo and in vitro models and has been documented as a human carcinogen, especially for leukemia [68,69]. Formaldehyde exposure was suggested to increase the levels of specific chromosome aberrations related to myeloid leukemia in the stem and progenitor cells, which are the targets for leukemogenesis [70]. In addition, a study on a mouse model demonstrated that aspartame could induce DNA strand breaks in bone marrow cells [22]. Apart from sugar alternatives, potential carcinogenic colors and flavorings in ASBs, or packaging materials of the beverage containers, might also increase the risk of leukemia, such as 4-methylimidazole used in cola drinks [71]. The dose of daily ASB intake is another issue that should be considered. The current acceptable daily intake of aspartame for humans is 40 mg/kg body weight in Europe and 50 mg/kg body weight in the United States. However, an animal trial reported a potential carcinogenic daily dose of 20 mg/kg body weight of aspartame intake, much less than the current criteria [24]. In our study, a small but significant linear dose–response relationship was found on the risk of leukemia with ASB intake. Per one serving (about 5 mg/kg body weight of total aspartame intake) of daily ASB intake increment was associated with about a 15% increase in the risk of leukemia, suggesting that the carcinogenic dose of ASB might be underestimated in current guidance.

For digestive system cancers, recent animal studies suggested that LCSs could affect gut microbiome status [72,73,74]. Several polyols added in ASBs, including isomaltose and maltitol, increased bifidobacterial numbers in healthy subjects and might have prebiotic actions [74,75,76]. Moreover, lactitol, a kind of LCS, was suggested to decrease the populations of Bacteroides [75]. Bacteroides can produce enterotoxins and reactive oxygen species and consequently cause oxidative DNA damage, induce inflammation, and damage the epithelial barrier and thus are involved in the development of colorectal cancer [77,78,79]. A recent cohort study also found that substituting one serving of SSBs per day with an equivalent amount of ASBs was associated with an 18.7% reduction in the incidence of proximal colon cancer [80]. However, it cannot be ignored that some LCSs also exhibit carcinogenic effects. For example, an in vitro study showed a pro-angiogenic effect and dose-dependent cytotoxic effect of aspartame in human colorectal carcinoma cells [21]. Owing to the limited number of included studies (*n* = 3) and lack of acknowledgment of the type of LCSs used in the ASBs, more studies are still needed to confirm the effect of ASB consumption on colorectal cancer risk.

Previous studies that suggested a higher consumption of SSBs rather than ASBs were associated with a larger increase in visceral adipose tissue volume [81]. Visceral fat is associated with alterations to immunological, metabolic, and endocrine function and with the risk of cancers [82]. A positive association between SSB intake and increased risk of obesity-related cancer has also been found in a prospective cohort study [39]. As for ASBs, some cellular and animal models found that frequent consumption of LCS might impair energy balance by suppressing GLP-1 release and inducing metabolic derangements [83,84]. In most epidemiologic studies, the positive association between LCS and obesity was supposed to be attributed to reverse causality [85]. Debras et al. found a positive association between ASB intake and the risk of obesity-related cancers, while a null association was shown in a previous study by Hodge et al. [38,39]. We grouped the 14 types of cancers involved in the 17 included cohorts according to the IARC guidelines [51] and found no evidence of the association between ASB intake and the risk of obesity-related cancers. Since the dietary habits might be distinct between individuals with or without obesity [86], we also conducted a subgroup analysis by baseline BMI. No significant association was found in either BMI ≥ 25 or BMI < 25 groups. Considering the positive association of SSBs with weight gain and the risk of obesity-related cancers [39], ASBs might serve as a healthier alternative to SSBs to prevent obesity-related cancers. However, the long-term health effects of high ASB consumption still need to be investigated in future studies.

According to the National Heart, Lung, and Blood Institute Growth and Health Study, the risk of early menarche was positively associated with a higher intake of aspartame [87]. Earlier onset of puberty was revealed to be a risk factor for breast and prostate cancers [88,89]. However, a null association was observed for ASB consumption and early menarche when BMI was included in the analyses [87]. In line with this, our results suggested no association between ASB intake and hormone-related cancer, with BMI as one of the prevalent adjustments in the included studies.

In the subgroup analysis stratified by area, the positive association of ASB intake with overall cancer risk was observed among Europeans, with the exception of Americans and Australians. The high ASB consumption among Europeans at recruitment may be one reason for this difference. Due to the limited number of studies (*n* = 3), more investigations among Europeans are needed for further verification. The risk of cancers also increased in studies that used lifestyle questionnaires instead of validated FFQs. The four studies that used lifestyle questionnaires recruited subjects among Europeans. In addition, we found a significant association between high ASB consumption and cancer risk in studies with three-category classification instead of further multiple-category classification of ASB consumption. A nonlinear model of ASB consumption with cancer risk might explain the results. However, our results should be further investigated due to the limited relevant studies and the insufficient study subjects in the included studies.

Our meta-analysis provides the most updated summary estimates of the associations, as well as the dose–response relationships between ASB intake and cancer incidence. The included studies were prospective cohort studies with large sample sizes and long-term follow-ups. According to sensitivity analyses, associations were robust. Nevertheless, some limitations should be addressed. Firstly, though the maximally adjusted *RR*s were adopted, residual or unmeasured confounding could not be ruled out completely. Higher ASB intake as a replacement for SSBs could serve as a marker of higher economic ability and a healthier diet. Incomplete adjustment for economic conditions could lead to an underestimation of the association between ASB exposure and cancer incidence. Secondly, the assessment of ASB intake was based on FFQ or lifestyle questionnaires, and information was lacking regarding the specific types of sweeteners consumed by the participants. Thirdly, the vast majority of our study population comprised white races. The generalizability of the results to other ethnicities remains to be explored. Fourthly, the available studies in some analyses were limited, and the certainty of evidence regarding site-specific cancers was mostly low or very low. Finally, moderate heterogeneity was detected in some outcomes. Subgroup analysis was conducted on overall cancer risk to explore the potential sources of heterogeneity in our meta-analysis. However, we did not conduct further subgroup analysis on site-specific cancers due to limited available studies. Further investigations are thus required to fully explain the heterogeneity in the future. Taken together, our results are required to be validated further in well-designed prospective studies and randomized controlled trials.

## 5. Conclusions

Our meta-analysis revealed, with moderate certainty of the evidence, that high levels of dietary ASB consumption might be associated with leukemia risk, and a per one serving (355 mL) increase in daily ASB intake was related to a 15% increase in the risk of leukemia. Furthermore, the results suggested a protective effect of ASB intake on the risk of colorectal cancer with low certainty of evidence. Given the low and moderate certainty of evidence and the limited number of included studies, further prospective cohort studies and randomized controlled trials are needed to expound the associations.

## Figures and Tables

**Table 1:** Characteristics of the studies included in the meta-analyses.

No.	Study, Year, Age (at Enrollment), (Sex), PMID ^*^	Study Cohort	Area	Follow-Up (Years)	Case/Participant	Cancer Type	Comparison Level (mL/d) (Lowest vs. Highest)	Adjustment for Confounders	Reference
1	Chazelas, 2019, 18–73, (Male and Female), PMID:31292122	The French NutriNet-Santé cohort	Europe	5.1	2193/101,257	Cancers including breast, colorectal, and prostate cancer	4.3 vs. 9.9, Quartile 1 vs. Quartile 4	Age; sex; energy intake without alcohol; sugar intake from other dietary sources; alcohol, sodium, lipid, and fruit and vegetable intakes; body mass index; height; physical activity; smoking status; number of 24-h dietary records; family history of cancer; educational level; the following prevalent conditions at baseline: type 2 diabetes, hypertension, major cardiovascular event, and dyslipidaemia; and additional adjustments for the number of biological children, menopausal status at baseline, hormonal treatment for menopause at baseline and during follow-up, and oral contraception use at baseline and during follow-up for breast cancer analyses.	[59]
2	Romanos-Nanclares, 2021, 30–55 (Female), PMID:34114021	The Nurses’ Health Study AND The Nurses’ Health Study II	US	26.48	7495/82,713 for NHS 3884/93,085 for NHSII	Breast cancer	<11.83 vs. ≥355, Quartile 1 vs. Quartile 4	Age; SSB intake; race; age at menarche; age at menopause; postmenopausal hormone use; oral contraceptive use history; parity and age at first birth; breastfeeding history; family history of breast cancer; history of benign breast disease; height; cumulatively updated alcohol intake; cumulatively updated total caloric intake; physical activity; BMI at age 18 years; modified Alternate Healthy Eating Index score (with SSBs and alcohol removed); socioeconomic status; and the change in weight since age 18.	[43]
3	Hur, 2021,25–42, (Female), PMID:33958435	The Nurses’ Health Study II	US	24	109/95,464	Colorectal cancer	<33.79 vs. ≥59.14, Quartile 1 vs. Quartile 4	Age; energy intake; race; height; body mass index; menopausal status and menopausal hormone use; family history of colorectal cancer; smoking status; physical activity; regular use of aspirin; regular use of non-steroidal anti-inflammatory drugs; current use of multivitamins; intake of alcohol, red and processed meat, and dietary fiber; total folate and total calcium; Alternative Healthy Eating Index-2010 score without sugar-sweetened beverages and alcohol; and lower endoscopy due to screening or for other indications within the past 10 years.	[42]
4	Debras, 2022, 28–57, (Male and Female), PMID: 35324894	The French NutriNet-Santé cohort	Europe	7.7	3358/102,865	All cancers, breast cancer, prostate cancer, and obesity-related cancers	0 vs. 207.77, Tertile 1 vs. Tertile 3	Age; sex; BMI; height; percentage weight gain during follow-up; physical activity; smoking status; number of smoked cigarettes in pack-years; educational level; number of 24-h dietary records; family history of cancer; prevalent diabetes; energy intake without alcohol; and daily intakes of alcohol, sodium, saturated fatty acids, fiber, sugar, fruits, vegetables, whole-grain foods, and dairy products. Breast cancer models were also adjusted for age at menarche, age at first child, number of biological children, baseline menopausal status, oral contraceptive use at baseline and during follow-up, and hormonal treatment for menopause at baseline and during follow-up.	[38]
5	Bassett, 2020, 27–76, (Male and Female), PMID:31693185	The Melbourne Collaborative Cohort Study	Australia	19	4789/35,109	Cancer not related to obesity including prostate, diffuse large B-cell lymphoma, noncardia gastric, lung, melanoma, premenopausal breast, bladder, brain, and lymphoid leukemia	<12.5 vs. >375, Quintile 1 vs. Quintile 5	Alcohol intake; country of birth; Mediterranean diet score; physical activity; socioeconomic position; sex and smoking status; and frequency of sugar-sweetened soft drink consumption.	[60]
6	Hodge, 2018, 27–76, (Male and Female), PMID:29463332	The Melbourne Collaborative Cohort Study	Australia	11.6	3283/35,593	Obesity-related cancers including aggressive prostate, ovary, kidney, colorectum, breast, endometrium, and gastric cardia cancer	<12.5 vs. >375, Quintile 1 vs. Quintile 5	Socio-Economic Indexes for Areas; country of birth; alcohol intake; smoking status; physical activity; sex; Mediterranean diet score; sugar-sweetened soft drink consumption; and waist circumference.	[39]
7	Navarrete-Muñoz, 2016, 25–42, (Male and Female), PMID:27510540	European Prospective Investigation into Cancer and Nutrition	Europe	11.6	865/477,199	Pancreatic cancer	0 vs. >92.2, Sextile 2 vs. Sextile 6	Center; sex; age at recruitment; educational level; physical activity; smoking status; alcohol consumption; juice, nectar, and soft drink consumption; energy intake; diabetes; and BMI.	[48]
8	McCullough, 2014, 47–95, (Male and Female), PMID:25342696	The Cancer Prevention Study-II Nutrition Cohort	US	10	1196/100,442	Non-Hodgkin lymphoma	0 vs. >355, Quintile 1 vs. Quintile 5	Age at baseline; gender; history of diabetes; BMI; smoking status; energy intake; and sugar-sweetened beverage intake.	[37]
9	Inoue-Choi, 2013, 52–71, (Female), PMID:24273064	The Iowa Women’s Health Study	US	18.79	595/23,039	Post-endometrial cancer: type I, type II	0 vs. 288, Quintile 1 vs. Quintile 5	Age; smoking status; physical activity; alcohol use; estrogen use; age at menarche; age at menopause; number of live births; coffee intake; and BMI.	[61]
10	Schernhammer, 2012, 30–55/40–75, (Male and Female), PMID:23097267	The Nurses’ Health Study AND The Health Professionals Follow-Up Study	US	17.88	888/47,810 for HPFUS 1060/77,218 for NHS	Hematopoietic cancers including non-Hodgkin lymphoma, multiple myeloma, and leukemia	0 vs. >355, Quintile 1 vs. Quintile 5	Age; questionnaire cycle; sugar-sweetened soda consumption; fruit and vegetable consumption; multivitamin use; intakes of alcohol, saturated fat, and animal protein; total energy; race; BMI; height; discretionary physical activity; smoking history; menopausal status and use of hormone replacement therapy (women only).	[36]
11	Bao, 2008, 50–71, (Male and Female), PMID:18689380	The NIH-AARP Diet and Health Study cohort	US	7.2	1258/487,922	Pancreatic cancer	0 vs. 816.9, Sextile 1 vs. Sextile 6	Age; sex; race; education; BMI; alcohol consumption; smoking status; physical activity; energy-adjusted red meat consumption; energy-adjusted folate consumption; total energy; and regular soft drink consumption.	[62]
12	Lim, 2006, 62.0, (Male and Female), PMID:16985027	The NIH-AARP Diet and Health Study cohort	US	5.2	2203/473,984	Hematopoietic cancers including Hodgkin lymphoma, multiple myeloma and lymphoid malignancies, gliomas	0 vs. 1296, Sextile 1 vs. Sextile 6	Age at entry; sex; ethnicity; BMI; and history of diabetes.	[35]
13	Schernhammer, 2005, 25–42, (Male and Female), PMID:16172216	The Nurses’ Health Study AND The Health Professionals Follow-up Study	US	15.37	174/49,364 for HPFUS 205/88,794 for NHS	Pancreatic cancer	<12 vs. >154, Tertile 1 vs. Tertile 3	Age; gender; follow-up cycle; history of diabetes; smoking status; quintiles of caloric intake; non-vigorous physical activity; and other soft drink consumption.	[63]
14	Zamora-Ros, 2022, 41.3–60.9, (Male and Female), PMID: 35907037	European Prospective Investigation into Cancer and Nutrition	Europe	14	495/450,064	Differentiated thyroid cancer	0 vs. 3411, Quartile 1 vs. Quartile 4	Sex; center; age at recruitment; BMI; smoking status; physical activity; educational level; alcohol and energy intake; and for women: menopausal status, oral contraceptive use, and infertility problems.	[44]

^*^: References [35,36,37,38,39,42,43,44,48,59,60,61,62,63].

**Table 2:** Subgroup analyses stratified by characteristics of study subjects, measurements of exposure, outcome assessment, and data analysis. Results were pooled with random-effect model. ASB, artificially sweetened beverage; BMI, body mass index; FFQ, Food Frequency Questionnaire; SSB, sugar-sweetened beverage.

Subgroup	Number of Studies	*RR* (95% CI)	*p*	*I*^2^ (%)	*p_-Heterogeneity_*	*tau* ^2^
**Characteristics of study subjects**						
Area						
North America	11	1.00 (0.91–1.09)	0.936	55.9%	0.012	0.011
Australia	2	1.12 (0.92–1.38)	0.254	44.7%	0.179	0.010
Europe	4	1.10 (1.01–1.19)	0.025	0.0%	0.446	0.000
Age at recruitment						
≥60	4	0.95 (0.81–1.12)	0.551	42.0%	0.160	0.012
<60	13	1.05 (0.97–1.14)	0.210	56.1%	0.007	0.009
Sex						
Female	7	0.95 (0.90–1.01)	0.085	0.0%	0.442	0.000
Male	3	1.15 (0.86–1.53)	0.344	64.9%	0.058	0.041
Sample size						
More than 50,000 participants	12	1.01 (0.95–1.07)	0.871	30.7%	0.146	0.003
Less than 50,000 participants	5	1.07 (0.87–1.32)	0.502	72.2%	0.006	0.039
Mean/median BMI (kg/m^2^) at recruitment						
≥25 and <30	10	1.05 (0.93–1.19)	0.430	57.7%	0.011	0.022
≥18 and <25	5	1.00 (0.92–1.10)	0.940	60.5%	0.038	0.005
Duration of follow-up						
Less than 10 years	4	1.07 (0.96–1.19)	0.245	36.8%	0.191	0.005
More than 10 years	13	1.02 (0.93–1.11)	0.701	53.2%	0.012	0.011
**Measurements of exposure**						
ASB measurement approach						
Validated FFQ	13	1.02 (0.93–1.11)	0.708	57.2%	0.005	0.012
Lifestyle questionnaires	4	1.10 (1.01–1.19)	0.025	0.0%	0.636	0.000
ASB measurement frequency						
Once, only at baseline	8	1.02 (0.91–1.15)	0.708	36.0%	0.141	0.010
Repeated measurements	9	1.04 (0.94–1.14)	0.466	65.3%	0.003	0.010
**Outcome assessment**						
Ascertainment of cancers						
Access to disease registry	8	1.06 (0.93–1.20)	0.404	30.7%	0.183	0.010
Self-report but with subsequent external validation	9	1.02 (0.93–1.11)	0.680	65.0%	0.004	0.009
**Data analysis**						
Levels of ASB consumption in controls						
High vs. non-consume	12	1.03 (0.94–1.12)	0.541	61.4%	0.003	0.011
High vs. low	5	1.05 (0.93–1.19)	0.441	19.0%	0.294	0.004
Classifications of ASB consumption						
Three-category	3	1.12 (1.02–1.22)	0.018	0.0%	0.521	0.000
Four-category	5	0.96 (0.91–1.02)	0.180	0.7%	0.402	0.000
Five-category	6	1.06 (0.90–1.25)	0.478	69.0%	0.006	0.028
Six-category	3	1.04 (0.84–1.28)	0.744	31.4%	0.233	0.011
Adjusted for SSB intake						
Yes	10	1.07 (0.95–1.20)	0.271	50.6%	0.033	0.018
No	7	0.99 (0.92–1.08)	0.893	52.4%	0.050	0.005
Adjusted for adiposity measures						
Yes	14	1.02 (0.94–1.10)	0.667	55.4%	0.006	0.009
No	3	1.15 (0.98–1.35)	0.093	5.5%	0.347	0.001

**Table 3:** Summary findings for grading the certainty of the evidence of artificially sweetened beverage (ASB) intake and cancer risk.

No. of Studies	Study Design	Certainty Assessment					*RR* (95% CI)	Certainty *
		Risk of Bias	Inconsistency	Indirectness	Imprecision	Other Considerations		
Breast cancer								
4	observational studies	moderate ^a^	serious ^b^	serious ^c^	not serious	none	0.99 (0.90–1.08)	⨁◯◯◯ VERY LOW
Colorectal cancer								
3	observational studies	moderate ^a^	not serious	serious ^c^	not serious	none	0.78 (0.62–0.99)	⨁⨁◯◯ LOW
Endometrial cancer								
2	observational studies	moderate ^a^	not serious	serious ^c^	serious ^d^	none	0.81 (0.64–1.03)	⨁◯◯◯ VERY LOW
Gastric cancer								
1	observational study	moderate ^a^	not serious	serious ^c^	serious ^d^	none	1.03 (0.53–1.99)	⨁◯◯◯ VERY LOW
Gliomas								
1	observational study	moderate ^a^	not serious	serious ^c^	serious ^d^	dose–response gradient	0.73 (0.46–1.15)	⨁⨁◯◯ LOW
Hodgkin lymphoma								
1	observational study	moderate ^a^	not serious	serious ^c^	serious ^d^	none	0.77 (0.45–1.33)	⨁◯◯◯ VERY LOW
Kidney cancer								
1	observational study	moderate ^a^	not serious	serious ^c^	serious ^d^	none	0.92 (0.46–1.84)	⨁◯◯◯ VERY LOW
Leukemia								
3	observational studies	moderate ^a^	not serious	serious ^c^	not serious	dose–response gradient	1.35 (1.03–1.77)	⨁⨁⨁◯ MODERATE
Multiple myeloma								
3	observational studies	moderate ^a^	serious ^e^	serious ^c^	serious ^d^	dose–response gradient	1.18 (0.69–2.02)	⨁◯◯◯ VERY LOW
Non-Hodgkin lymphoma								
4	observational studies	moderate ^a^	not serious	serious ^c^	not serious	none	1.05 (0.91–1.21)	⨁⨁◯◯ LOW
Ovarian cancer								
1	observational study	moderate ^a^	not serious	serious ^c^	serious ^d^	none	1.37 (0.72–2.61)	⨁◯◯◯ VERY LOW
Pancreatic cancer								
4	observational studies	moderate ^a^	not serious	serious ^c^	serious ^d^	none	1.10 (0.92–1.31)	⨁◯◯◯ VERY LOW
Prostate cancer								
2	observational studies	moderate ^a^	serious ^f^	serious ^c^	serious ^d^	none	1.06 (0.70–1.62)	⨁◯◯◯ VERY LOW
Thyroid cancer								
1	observational study	moderate ^a^	not serious	serious ^c^	serious ^d^	none	1.16 (0.80–1.69)	⨁◯◯◯ VERY LOW
All cancer								
17	observational studies	moderate ^a^	serious ^g^	serious ^c^	not serious	none	1.03 (0.96–1.11)	⨁◯◯◯ VERY LOW

* All outcomes started with high-quality evidence. The five reasons for rating down the quality of evidence were as follows: Risk of bias (evaluated by ROBINS-E)—low risk of bias: do not downgrade, moderate risk of bias: downgrade one level, serious risk of bias: downgrade two levels, critical risk of bias: downgrade three levels. Inconsistency—we considered rating down for inconsistency when heterogeneity or large among-study difference was tested (*p_-heterogeneity_* < 0.10 or *I*^2^ ≥ 50.0%), or point estimates varied widely across studies, or the confidence intervals (CIs) showed minimal or no overlap. Indirectness—rated down if there were indirect factors that might limit the generalizability of the results. These factors might be related to target populations, interventions, and outcome measurements. Imprecision—precision was adequate if the number of patients or events exceeded the optimal information size (OIS) criterion (at least 800 (400 per group)), and the 95% CI excluded no effect (i.e., CI around *RR* excluded 1.0). If the OIS criterion was not met, the study was rated down for imprecision unless the sample size was very large. We also considered rating down for imprecision if CI failed to exclude appreciable benefit or harm (an *RR* under 0.75 or *RR* over 1.25) and CI overlapped no effect (i.e., CI includes *RR* of 1.0) even if the OIS criterion was met. Publication bias (evaluated by Begg’s test and Egger’s test)—Certainty of evidence was downgraded if there was potential publication bias (*p*_-begg_ < 0.100 or *p*_-egger_ < 0.100). The factors used to upgrade the certainty of evidence included a large magnitude of association, a dose–response gradient, and attenuation of plausible confounding.
Downgraded by one level. There were one or more studies at moderate risk of bias. Downgraded by one level. *I*^2^ = 55.90%, *p* = 0.079, and the results showed different point estimates and minimal overlapping CIs. Downgraded by one level. The intake of ASB was mostly assessed by questionnaires, which could not directly reflect the actual intake. Downgraded by one level. Serious imprecision was evaluated since although the OIS was met (more than 800 (400 per group)) and the 95% CI overlapped no effect (CI includes *RR* of 1.0), the CI failed to exclude important benefit or important harm (an *RR* reduction or *RR* increase of 25% or more) Downgraded by one level. *I*^2^ = 68.90%, *p* = 0.040, and the results showed different point estimates and minimal overlapping CIs. Downgraded by one level. *I*^2^ = 55.50%, *p* = 0.054, and the results showed different point estimates and minimal overlapping CIs. Downgraded by one level. *I*^2^ = 53.0%, *p* = 0.005, and the results showed different point estimates and minimal overlapping CIs.

## Data Availability

Data for the meta-analysis are available in the manuscript’s tables. All statistical analyses were undertaken using STATA/MP, version 14.0 (Stata Corporation, College Station, TX, USA).

## Supplementary Materials

The following supporting information can be downloaded at: https://www.mdpi.com/article/10.3390/nu14214445/s1, Figure S1. Dose–response analysis of artificially sweetened beverage (ASB) intake with risk of cancers; Figure S2. Forest plot of artificially sweetened beverage (ASB) intake and risk of site-specific cancers; Figure S3. Sensitivity analysis of studies included in the meta-analysis on ASB intake and overall cancer risk; Figure S4. Funnel plots of studies included in the meta-analysis. Table S1. Criteria adopted for Risk of Bias (ROB) assessment; Table S2. Detailed information for Risk of Bias (ROB) assessment of included studies; Table S3. Summary Risk of Bias (ROB) assessment with overall study-level risk of bias; Table S4. Minimally and maximally adjusted *RR* by cancer type.

## References

[1] Zhou M., Wang H., Zeng X., Yin P., Zhu J., Chen W., Li X., Wang L., Liu Y., Liu J. (2019). Mortality, morbidity, and risk factors in China and its provinces, 1990–2017: A systematic analysis for the Global Burden of Disease Study 2017. Lancet.

[2] Malik V.S., Hu F.B. (2022). The role of sugar-sweetened beverages in the global epidemics of obesity and chronic diseases. Nat. Rev. Endocrinol..

[3] Atkinson F.S., Brand-Miller J.C., Foster-Powell K., Buyken A.E., Goletzke J. (2021). International tables of glycemic index and glycemic load values 2021: A systematic review. Am. J. Clin. Nutr..

[4] Jenkins D.J., Wolever T.M., Taylor R.H., Barker H., Fielden H., Baldwin J.M., Bowling A.C., Newman H.C., Jenkins A.L., Goff D.V. (1981). Glycemic index of foods: A physiological basis for carbohydrate exchange. Am. J. Clin. Nutr..

[5] Augustin L.S.A., Kendall C.W.C., Jenkins D.J.A., Willett W.C., Astrup A., Barclay A.W., Björck I., Brand-Miller J.C., Brighenti F., Buyken A.E. (2015). Glycemic index, glycemic load and glycemic response: An International Scientific Consensus Summit from the International Carbohydrate Quality Consortium (ICQC). Nutr. Metab. Cardiovasc. Dis..

[6] Bell S.J., Sears B. (2003). Low-glycemic-load diets: Impact on obesity and chronic diseases. Crit. Rev. Food Sci. Nutr..

[7] Brand-Miller J.C. (2003). Glycemic load and chronic disease. Nutr. Rev..

[8] Long T., Liu K., Long J., Li J., Cheng L. (2022). Dietary glycemic index, glycemic load and cancer risk: A meta-analysis of prospective cohort studies. Eur. J. Nutr..

[9] Schwingshackl L., Schwedhelm C., Hoffmann G., Knüppel S., Iqbal K., Andriolo V., Bechthold A., Schlesinger S., Boeing H. (2017). Food Groups and Risk of Hypertension: A Systematic Review and Dose-Response Meta-Analysis of Prospective Studies. Adv. Nutr..

[10] Malik V.S., Pan A., Willett W.C., Hu F.B. (2013). Sugar-sweetened beverages and weight gain in children and adults: A systematic review and meta-analysis. Am. J. Clin. Nutr..

[11] Imamura F., O’Connor L., Ye Z., Mursu J., Hayashino Y., Bhupathiraju S.N., Forouhi N.G. (2015). Consumption of sugar sweetened beverages, artificially sweetened beverages, and fruit juice and incidence of type 2 diabetes: Systematic review, meta-analysis, and estimation of population attributable fraction. BMJ-Br. Med. J..

[12] Li Y., Guo L., He K., Huang C., Tang S. (2021). Consumption of sugar-sweetened beverages and fruit juice and human cancer: A systematic review and dose-response meta-analysis of observational studies. J. Cancer.

[13] Micha R., Penalvo J.L., Cudhea F., Imamura F., Rehm C.D., Mozaffarian D. (2017). Association Between Dietary Factors and Mortality from Heart Disease, Stroke, and Type 2 Diabetes in the United States. Jama-J. Am. Med. Assoc..

[14] Sylvetsky A.C., Rother K.I. (2016). Trends in the consumption of low-calorie sweeteners. Physiol. Behav..

[15] Rother K.I., Conway E.M., Sylvetsky A.C. (2018). How Non-nutritive Sweeteners Influence Hormones and Health. Trends Endocrinol. Metab..

[16] Nakagawa Y., Nagasawa M., Yamada S., Hara A., Mogami H., Nikolaev V.O., Lohse M.J., Shigemura N., Ninomiya Y., Kojima I. (2009). Sweet taste receptor expressed in pancreatic beta-cells activates the calcium and cyclic AMP signaling systems and stimulates insulin secretion. PLoS ONE.

[17] Duffy V.B., Anderson G.H. (1998). Position of the American Dietetic Association: Use of nutritive and nonnutritive sweeteners. J. Am. Diet. Assoc..

[18] Fakhouri T.H., Kit B.K., Ogden C.L. (2012). Consumption of diet drinks in the United States, 2009–2010. NCHS Data Brief.

[19] Suez J., Korem T., Zeevi D., Zilberman-Schapira G., Thaiss C.A., Maza O., Israeli D., Zmora N., Gilad S., Weinberger A. (2014). Artificial sweeteners induce glucose intolerance by altering the gut microbiota. Nature.

[20] Qin P., Li Q., Zhao Y., Chen Q., Sun X., Liu Y., Li H., Wang T., Chen X., Zhou Q. (2020). Sugar and artificially sweetened beverages and risk of obesity, type 2 diabetes mellitus, hypertension, and all-cause mortality: A dose-response meta-analysis of prospective cohort studies. Eur. J. Epidemiol..

[21] Maghiari A.L., Coricovac D., Pinzaru I.A., Macașoi I.G., Marcovici I., Simu S., Navolan D., Dehelean C. (2020). High Concentrations of Aspartame Induce Pro-Angiogenic Effects in Ovo and Cytotoxic Effects in HT-29 Human Colorectal Carcinoma Cells. Nutrients.

[22] Bandyopadhyay A., Ghoshal S., Mukherjee A. (2008). Genotoxicity testing of low-calorie sweeteners: Aspartame, acesulfame-K., and saccharin. Drug Chem. Toxicol..

[23] Alleva R., Borghi B., Santarelli L., Strafella E., Carbonari D., Bracci M., Tomasetti M. (2011). In vitro effect of aspartame in angiogenesis induction. Toxicol. Vitr..

[24] Soffritti M., Belpoggi F., Degli Esposti D., Lambertini L., Tibaldi E., Rigano A. (2006). First experimental demonstration of the multipotential carcinogenic effects of aspartame administered in the feed to Sprague-Dawley rats. Environ. Health Perspect..

[25] Soffritti M., Padovani M., Tibaldi E., Falcioni L., Manservisi F., Lauriola M., Bua L., Manservigi M., Belpoggi F. (2016). Sucralose administered in feed, beginning prenatally through lifespan, induces hematopoietic neoplasias in male swiss mice. Int. J. Occup. Environ. Health.

[26] Ishii H. (1981). Incidence of brain tumors in rats fed aspartame. Toxicol. Lett..

[27] Jeffrey A.M., Williams G.M. (2000). Lack of DNA-damaging activity of five non-nutritive sweeteners in the rat hepatocyte/DNA repair assay. Food Chem. Toxicol..

[28] Bucher Bristol J.R., French D.W., Hailey J.E., Haseman J.R., Herbert J.K., Malarkey R.A., Maronpot D.E., Peckham R.R., Roycroft J.C. (2005). NTP report on the toxicology studies of aspartame (CAS No. 22839-47-0) in genetically modified (FVB Tg.AC hemizygous) and B6.129-Cdkn2atm1Rdp (N2) deficient mice and carcinogenicity studies of aspartame in genetically modified. Natl. Toxicol. Program Genet. Modif. Model. Rep..

[29] Sullivan J.W. (1982). Epidemiologic survey of bladder cancer in greater New Orleans. J. Urol..

[30] Sturgeon S.R., Hartge P., Silverman D.T., Kantor A.F., Linehan W.M., Lynch C., Hoover R.N. (1994). Associations between bladder cancer risk factors and tumor stage and grade at diagnosis. Epidemiology.

[31] Wang J., Wu X., Kamat A., Barton Grossman H., Dinney C.P., Lin J. (2013). Fluid intake, genetic variants of UDP-glucuronosyltransferases, and bladder cancer risk. Br. J. Cancer.

[32] Chan J.M., Wang F., Holly E.A. (2009). Sweets, sweetened beverages, and risk of pancreatic cancer in a large population-based case-control study. Cancer Causes Control..

[33] Murtaugh M.A., Ma K.N., Caan B.J., Slattery M.L. (2004). Association of fluids from beverages with risk of rectal cancer. Nutr. Cancer.

[34] Ibiebele T.I., Hughes M.C., O’Rourke P., Webb P.M., Whiteman D.C. (2008). Cancers of the esophagus and carbonated beverage consumption: A population-based case-control study. Cancer Causes Control..

[35] Lim U., Subar A.F., Mouw T., Hartge P., Morton L.M., Stolzenberg-Solomon R., Campbell D., Hollenbeck A.R., Schatzkin A. (2006). Consumption of aspartame-containing beverages and incidence of hematopoietic and brain malignancies. Cancer Epidemiol. Biomark. Prev..

[36] Schernhammer E.S., Bertrand K.A., Birmann B.M., Sampson L., Willett W.C., Feskanich D. (2012). Consumption of artificial sweetener- and sugar-containing soda and risk of lymphoma and leukemia in men and women. Am. J. Clin. Nutr..

[37] McCullough M.L., Teras L.R., Shah R., Diver W.R., Gaudet M.M., Gapstur S.M. (2014). Artificially and sugar-sweetened carbonated beverage consumption is not associated with risk of lymphoid neoplasms in older men and women. J. Nutr..

[38] Debras C., Chazelas E., Srour B., Druesne-Pecollo N., Esseddik Y., Szabo de Edelenyi F., Agaësse C., de Sa A., Lutchia R., Gigandet S. (2022). Artificial sweeteners and cancer risk: Results from the NutriNet-Santé population-based cohort study. PLoS Med..

[39] Hodge A.M., Bassett J.K., Milne R.L., English D.R., Giles G.G. (2018). Consumption of sugar-sweetened and artificially sweetened soft drinks and risk of obesity-related cancers. Public Health Nutr..

[40] Llaha F., Gil-Lespinard M., Unal P., de Villasante I., Castañeda J., Zamora-Ros R. (2021). Consumption of Sweet Beverages and Cancer Risk. A Systematic Review and Meta-Analysis of Observational Studies. Nutrients.

[41] Heath A.K., Clasen J.L., Jayanth N.P., Jenab M., Tjonneland A., Petersen K.E.N., Overvad K., Srour B., Katzke V., Bergmann M.M. (2021). Soft Drink and Juice Consumption and Renal Cell Carcinoma Incidence and Mortality in the European Prospective Investigation into Cancer and Nutrition. Cancer Epidemiol. Biomark. Prev..

[42] Hur J., Otegbeye E., Joh H.K., Nimptsch K., Ng K., Ogino S., Meyerhardt J.A., Chan A.T., Willett W.C., Wu K. (2021). Sugar-sweetened beverage intake in adulthood and adolescence and risk of early-onset colorectal cancer among women. Gut.

[43] Romanos-Nanclares A., Collins L.C., Hu F.B., Willett W.C., A Rosner B., Toledo E., Eliassen A.H. (2021). Sugar-Sweetened Beverages, Artificially Sweetened Beverages, and Breast Cancer Risk: Results From 2 Prospective US Cohorts. J. Nutr..

[44] Zamora-Ros R., Cayssials V., Clèries R., Torrents M., Byrnes G., Weiderpass E., Sandström M., Almquist M., Boutron-Ruault M.-C., Tjønneland A. (2022). Sweetened beverages are associated with a higher risk of differentiated thyroid cancer in the EPIC cohort: A dietary pattern approach. Eur. J. Nutr..

[45] Page M.J., McKenzie J.E., Bossuyt P.M., Boutron I., Hoffmann T.C., Mulrow C.D., Shamseer L., Tetzlaff J.M., Akl E.A., Brennan S.E. (2021). The PRISMA 2020 statement: An updated guideline for reporting systematic reviews. BMJ.

[46] Aune D., Greenwood D.C., Chan D.S.M., Vieira R., Vieira A.R., Navarro Rosenblatt D.A., Cade J., Burley V., Norat T. (2012). Body mass index, abdominal fatness and pancreatic cancer risk: A systematic review and non-linear dose-response meta-analysis of prospective studies. Ann. Oncol..

[47] Rong Y., Chen L., Zhu T., Song Y., Yu M., Shan Z., Sands A., Hu F.B., Liu L. (2013). Egg consumption and risk of coronary heart disease and stroke: Dose-response meta-analysis of prospective cohort studies. BMJ.

[48] Navarrete-Muñoz E.M., Wark P.A., Romaguera D., Bhoo-Pathy N., Michaud D., Molina-Montes E., Tjønneland A., Olsen A., Overvad K. (2016). Sweet-beverage consumption and risk of pancreatic cancer in the European Prospective Investigation into Cancer and Nutrition (EPIC). Am. J. Clin. Nutr..

[49] Stepien M., Duarte-Salles T., Fedirko V., Trichopoulou A., Lagiou P., Bamia C., Overvad K., Tjønneland A., Hansen L., Boutron-Ruault M.-C. (2016). Consumption of soft drinks and juices and risk of liver and biliary tract cancers in a European cohort. Eur. J. Nutr..

[50] Sylvetsky A., Rother K.I., Brown R. (2011). Artificial sweetener use among children: Epidemiology, recommendations, metabolic outcomes, and future directions. Pediatr. Clin. North Am..

[51] Lauby-Secretan B., Scoccianti C., Loomis D., Grosse Y., Bianchini F., Straif K. (2016). Body Fatness and Cancer—Viewpoint of the IARC Working Group. N. Engl. J. Med..

[52] Morgan R.L., Thayer K.A., Santesso N., Holloway A.C., Blain R., Eftim S.E., Goldstone A.E., Ross P., Ansari M., Akl E.A. (2019). A risk of bias instrument for non-randomized studies of exposures: A users’ guide to its application in the context of GRADE. Environ. Int..

[53] Guyatt G.H., Oxman A.D., Vist G.E., Kunz R., Falck-Ytter Y., Alonso-Coello P., Schünemann H.J. (2008). GRADE: An emerging consensus on rating quality of evidence and strength of recommendations. BMJ.

[54] Higgins J.P., Thompson S.G., Deeks J.J., Altman D.G. (2003). Measuring inconsistency in meta-analyses. BMJ.

[55] Orsini N., Li R., Wolk A., Khudyakov P., Spiegelman D. (2012). Meta-analysis for linear and nonlinear dose-response relations: Examples, an evaluation of approximations, and software. Am. J. Epidemiol..

[56] Harrell F.E., Lee K.L., Pollock B.G. (1988). Regression models in clinical studies: Determining relationships between predictors and response. J. Natl. Cancer Inst..

[57] Egger M., Davey Smith G., Schneider M., Minder C. (1997). Bias in meta-analysis detected by a simple, graphical test. BMJ.

[58] Begg C.B., Mazumdar M. (1994). Operating characteristics of a rank correlation test for publication bias. Biometrics.

[59] Chazelas E., Srour B., Desmetz E., Kesse-Guyot E., Julia C., Deschamps V., Druesne-Pecollo N., Galan P., Hercberg S., Latino-Martel P. (2019). Sugary drink consumption and risk of cancer: Results from NutriNet-Santé prospective cohort. Bmj.

[60] Bassett J.K., Milne R.L., English D.R., Giles G.G., Hodge A.M. (2020). Consumption of sugar-sweetened and artificially sweetened soft drinks and risk of cancers not related to obesity. Int. J. Cancer.

[61] Inoue-Choi M., Robien K., Mariani A., Cerhan J.R., Anderson K.E. (2013). Sugar-sweetened beverage intake and the risk of type I and type II endometrial cancer among postmenopausal women. Cancer Epidemiol. Biomark. Prev..

[62] Bao Y., Stolzenberg-Solomon R., Jiao L., Silverman D.T., Subar A.F., Park Y., Leitzmann M.F., Hollenbeck A., Schatzkin A., Michaud D.S. (2008). Added sugar and sugar-sweetened foods and beverages and the risk of pancreatic cancer in the National Institutes of Health-AARP Diet and Health Study. Am. J. Clin. Nutr..

[63] Schernhammer E.S., Hu F.B., Giovannucci E., Michaud D.S., Colditz G.A., Stampfer M.J., Fuchs C.S. (2005). Sugar-sweetened soft drink consumption and risk of pancreatic cancer in two prospective cohorts. Cancer Epidemiol. Biomark. Prev..

[64] Soffritti M., Belpoggi F., Tibaldi E., Esposti D.D., Lauriola M. (2007). Life-span exposure to low doses of aspartame beginning during prenatal life increases cancer effects in rats. Environ. Health Perspect..

[65] Soffritti M., Belpoggi F., Manservigi M., Tibaldi E., Lauriola M., Falcioni L., Bua L. (2010). Aspartame administered in feed, beginning prenatally through life span, induces cancers of the liver and lung in male Swiss mice. Am. J. Ind. Med..

[66] Landrigan P.J., Straif K. (2021). Aspartame and cancer—New evidence for causation. Environ. Health.

[67] Tsang W.S., Clarke M.A., Parrish F.W. (1985). Determination of aspartame and its breakdown products in soft drinks by reverse-phase chromatography with UV detection. J. Agric. Food Chem..

[68] World Health Organization (2006). Formaldehyde, 2-Butoxyethanoland 1-Tert-Butoxypropan-2-ol: Summary of Data Reported and Evaluation.

[69] Report F., Backgrou C., Carcinog R., Doc B., Pubmed P.M. (2010). Final Report on Carcinogens Background Document for Formaldehyde. Rep. Carcinog. Backgr. Doc..

[70] Zhang L., Tang X., Rothman N., Vermeulen R., Ji Z., Shen M., Qiu C., Guo W., Liu S., Reiss B. (2010). Occupational exposure to formaldehyde, hematotoxicity, and leukemia-specific chromosome changes in cultured myeloid progenitor cells. Cancer Epidemiol. Biomark. Prev..

[71] Smith T.J.S., Wolfson J.A., Jiao D., Crupain M.J., Rangan U., Sapkota A., Bleich S.N., Nachman K.E. (2015). Caramel color in soft drinks and exposure to 4-methylimidazole: A quantitative risk assessment. PLoS ONE.

[72] Palmnäs M.S.A., Cowan T.E., Bomhof M.R., Su J., Reimer R.A., Vogel H.J., Hittel D.S., Shearer J. (2014). Low-dose aspartame consumption differentially affects gut microbiota-host metabolic interactions in the diet-induced obese rat. PLoS ONE.

[73] Lobach A.R., Roberts A., Rowland I.R. (2019). Assessing the in vivo data on low/no-calorie sweeteners and the gut microbiota. Food Chem. Toxicol..

[74] Ruiz-Ojeda F.J., Plaza-Díaz J., Sáez-Lara M.J., Gil A. (2019). Effects of Sweeteners on the Gut Microbiota: A Review of Experimental Studies and Clinical Trials. Adv. Nutr..

[75] Finney M., Smullen J., Foster H.A., Brokx S., Storey D.M. (2007). Effects of low doses of lactitol on faecal microflora, pH, short chain fatty acids and gastrointestinal symptomology. Eur. J. Nutr..

[76] Beards E., Tuohy K., Gibson G. (2010). A human volunteer study to assess the impact of confectionery sweeteners on the gut microbiota composition. Br. J. Nutr..

[77] Goodwin A.C., Destefano Shields C.E., Wu S., Huso D.L., Wu X., Murray-Stewart T.R., Hacker-Prietz A., Rabizadeh S., Woster P.M., Sears C.L. (2011). Polyamine catabolism contributes to enterotoxigenic Bacteroides fragilis-induced colon tumorigenesis. Proc. Natl. Acad. Sci. USA.

[78] Wu N., Yang X., Zhang R., Li J., Xiao X., Hu Y., Chen Y., Yang F., Lu N., Wang Z. (2013). Dysbiosis signature of fecal microbiota in colorectal cancer patients. Microb. Ecol..

[79] Saus E., Iraola-Guzmán S., Willis J.R., Brunet-Vega A., Gabaldón T. (2019). Microbiome and colorectal cancer: Roles in carcinogenesis and clinical potential. Mol. Aspects Med..

[80] Yuan C., Joh H.-K., Wang Q.-L., Zhang Y., A Smith-Warner S., Wang M., Song M., Cao Y., Zhang X., Zoltick E.S. (2022). Sugar-sweetened beverage and sugar consumption and colorectal cancer incidence and mortality according to anatomic subsite. Am. J. Clin. Nutr..

[81] Ma J., McKeown N.M., Hwang S.J., Hoffmann U., Jacques P.F., Fox C.S. (2016). Sugar-Sweetened Beverage Consumption Is Associated with Change of Visceral Adipose Tissue Over 6 Years of Follow-Up. Circulation.

[82] Doyle S.L., Donohoe C.L., Lysaght J., Reynolds J.V. (2012). Visceral obesity, metabolic syndrome, insulin resistance and cancer. Proc. Nutr. Soc..

[83] Swithers S.E. (2013). Artificial sweeteners produce the counterintuitive effect of inducing metabolic derangements. Trends Endocrinol. Metab..

[84] Swithers S.E., Laboy A.F., Clark K., Cooper S., Davidson T.L. (2012). Experience with the high-intensity sweetener saccharin impairs glucose homeostasis and GLP-1 release in rats. Behav. Brain Res..

[85] Rogers P.J., Hogenkamp P.S., de Graaf C., Higgs S., Lluch A., Ness A.R., Penfold C., Perry R., Putz P., Yeomans M.R. (2016). Does low-energy sweetener consumption affect energy intake and body weight? A systematic review, including meta-analyses, of the evidence from human and animal studies. Int. J. Obes..

[86] Grech A., Kam C.O., Gemming L., Rangan A. (2018). Diet-Quality and Socio-Demographic Factors Associated with Non-Nutritive Sweetener Use in the Australian Population. Nutrients.

[87] Mueller N.T., Jacobs D.R., MacLehose R.F., Demerath E.W., Kelly S.P., Dreyfus J.G., Pereira M.A. (2015). Consumption of caffeinated and artificially sweetened soft drinks is associated with risk of early menarche. Am. J. Clin. Nutr..

[88] Janghorbani M., Mansourian M., Hosseini E. (2014). Systematic review and meta-analysis of age at menarche and risk of type 2 diabetes. Acta Diabetol..

[89] Charalampopoulos D., McLoughlin A., Elks C.E., Ong K.K. (2014). Age at menarche and risks of all-cause and cardiovascular death: A systematic review and meta-analysis. Am. J. Epidemiol..

