# Supplementary material for: Artificially Sweetened Beverage Consumption and Cancer Risk: A Comprehensive Dose–Response Meta-Analysis of Prospective Studies
Source: Nutrients. 2022 Oct 22;14(21):4445. doi: 10.3390/nu14214445 (PMC9658995; doi:10.3390/nu14214445)
Supplement: Supplementary file 1 [file nutrients-14-04445-s001.zip › nutrients-1962526-supplementary.pdf]

nutrients

MDPI

Supplementary Table S1. Criteria adopted for Risk of Bias (ROB) assessment.

Domains	Criteria
Bias due to confounding	To be considered at low risk of bias, a study must include age, body mass index, smoking status, and energy intake/physical activity. The study will be assessed as serious ROB if there is no adjustment for more than one major confounder mentioned above.
Bias in selecting participants for the study	The selection of eligible participants must not be related to ASB exposure.
Bias in exposure classification	Studies are considered at low risk of bias if exposure classification was performed using the measurement of a biological specimen (e.g., blood, urine) at the beginning of the study. Studies are considered at moderate risk of bias if exposure assessment was performed using measurement by FFQs. Studies are considered at serious risk of bias if they rely on self-report for exposure classification or if criteria are not reported.
Bias in departure from intended exposure	There should be no concern about the departure from intended exposure due to the long term that subjects may have changed exposure during follow-up. A threshold of 20 years of mean follow-up has been considered at moderate risk for a possible change in exposure from the beginning of the study to the end of the follow-up.
Bias due to missing data	The reasons for exclusion were documented and unlikely to differ across the exposure threshold. Analyses were conducted that addressed loss to follow-up and are likely to have removed any risk of bias.
Bias in outcome measurement	Studies are considered at low risk of bias if outcome assessment is based on access to disease registry. Studies are considered at moderate risk of bias if outcome assessment is based on self-report but with subsequent external validation. Studies are considered at high risk of bias if outcome assessment is based on self-report only without external validation.
Bias in selection of reported results	Evidence that results have not been selected. Clear reporting of statistical methods.
Assessment of overall ROB	If at least one domain was found at serious ROB, the overall risk was considered serious. If more than one domain was found at moderate ROB, the overall risk was considered moderate. If all domains were at low ROB, the overall risk was considered low.

Abbreviation: ROB, Risk of Bias; ASB, artificially sweetened beverage; FFQ, Food Frequency Questionnaire.

Supplementary Table S2. Detailed information for Risk of Bias (ROB) assessment of included studies.

Studies, bias item	Risk of bias	Rationale
Bao (2008) [1]		
Bias due to confounding	Low	Model adjusted for age, sex, race, education, BMI, alcohol, smoking, physical activity, energy-adjusted red meat consumption, energy-adjusted folate consumption, total energy, and regular soft drink consumption.
Bias in selection of participants for the study	Low	Participant selection for the cohort was not related to the exposure or outcome.
Bias in measurement of exposures	Moderate	Exposure assessment was performed using measurement by FFQs.
Bias due to departures from intended exposures	Low	Dietary intakes were derived from the baseline 124-item FFQ. Follow-up less than 20 years.
Bias due to missing data	Low	Reasons for exclusion were documented and unlikely to differ across exposures threshold. Although some subjects were lost to follow-up and the missing data were not described by exposure status, the study authors conducted analyses that addressed loss to follow-up and are likely to have removed any risk of bias and thus judged a low risk of bias.
Bias in measurement of outcomes	Low	Incident cases of pancreatic cancer through December 31, 2003, were identified through the 11 state cancer registries.
Bias in selection of the reported result	Low	Study results were fully reported, including baseline and covariates distribution and stratified analyses according to many confounding factors. Statistical methods reported in the methods section were used and presented in the results.
Overall bias	Moderate	One domain was found at moderate ROB.
Bassett (2020) [2]		

Bias due to confounding	Moderate	Model adjusted for alcohol intake, country of birth, Mediterranean diet score, physical activity, socioeconomic position, sex and smoking status, and frequency of sugar-sweetened soft drink consumption. No adjustment for age (the reason for moderate ROB).
Bias in selection of participants for the study	Low	Participant selection for the cohort was not related to the exposure or outcome.
Bias in classification of exposures	Moderate	Exposure assessment was performed using measurement by FFQs.
Bias due to departures from intended exposures	Low	At baseline, participants completed a 121-item FFQ, including separate questions about the number of times in the past year they had consumed regular (sugar-sweetened) or diet (artificially sweetened) soft drinks. Follow-up less than 20 years.
Bias due to missing data	Moderate	Reasons for exclusion were documented and unlikely to differ across exposures threshold. The study authors did not conduct analyses that addressed loss to follow-up and thus judged a moderate risk of bias.
Bias in measurement of outcomes	Low	Incident cancer cases were ascertained from the Victorian.
Bias in selection of the reported result	Low	Cancer Registry or the Australian Cancer Database.
Overall bias	Moderate	Three domains were found at moderate ROB.
Chazelas (2019) [3]		

Bias due to confounding	Low	Model adjusted for age; sex; energy intake without alcohol; sugar intake from other dietary sources; alcohol, sodium, lipid, and fruit and vegetable intakes; body mass index; height; physical activity; smoking status; number of 24-hour dietary records; family history of cancer; educational level; the following prevalent conditions at baseline: type 2 diabetes, hypertension, major cardiovascular event, and dyslipidaemia; and additional adjustments for the number of biological children, menopausal status at baseline, hormonal treatment for menopause at baseline and during follow-up, and oral contraception use at baseline and during follow-up for breast cancer analyses.
Bias in selection of participants for the study	Low	Participant selection for the cohort was not related to the exposure or outcome.
Bias in classification of exposures	Moderate	Participants completed a set of five reported questionnaires.
Bias due to departures from intended exposures	Low	Dietary data were collected at baseline and every six months (to vary the season of completion). Participants were asked to fill three non-consecutive validated web-based 24-hour dietary records, randomly assigned over a two-week period. Follow-up less than 20 years.
Bias due to missing data	NI	No mention of missing data.
Bias in measurement of outcomes	Moderate	Included self-reported cancers, and 95% had a confirmed medical record.
Bias in selection of the reported result	Low	Study results were fully reported, including baseline and covariates distribution and stratified analyses according to many confounding factors. Statistical methods reported in the methods section were used and presented in the results.
Overall bias	Moderate	Two domains were found at moderate ROB.
Debras (2022) [4]		

Bias due to confounding	Low	Model adjusted for age; sex; BMI; height; percentage weight gain during follow-up; physical activity; smoking status; number of smoked cigarettes in pack-years; educational level; number of 24-hour dietary records; family history of cancer; prevalent diabetes; energy intake without alcohol; and daily intakes of alcohol, sodium, saturated fatty acids, fiber, sugar, fruit and vegetables, whole-grain foods, and dairy products. Breast cancer models were also adjusted for age at menarche, age at first child, number of biological children, baseline menopausal status, oral contraceptive use at baseline and during follow-up, and hormonal treatment for menopause at baseline and during follow-up.
Bias in selection of participants for the study	Low	Participant selection for the cohort was not related to the exposure or outcome.
Bias in classification of exposures	Moderate	Participants completed country-specific validated dietary questionnaires at baseline.
Bias due to departures from intended exposures	Low	Detailed information was collected at baseline and every year thereafter through a 5-questionnaire kit.
Bias due to missing data	Moderate	Reasons for exclusion were documented and unlikely to differ across exposures threshold. The study authors did not conduct analyses that addressed loss to follow-up and thus judged a moderate risk of bias.
Bias in measurement of outcomes	Moderate	Participants were asked to report all medications, treatments, and major health events on the annual health questionnaire (a specific check-up questionnaire) and were contacted by a physician of the research team to provide any relevant medical and anatomopathological reports.
Bias in selection of the reported result	Low	Study results were fully reported, including baseline and covariates distribution and stratified analyses according to many confounding factors. Statistical methods reported in the methods section were used and presented in the results.

Overall bias	Moderate	Three domains were found at moderate ROB.
Hodge (2018) [5]		
Bias due to confounding	Moderate	Model adjusted for Socio-Economic Indexes for Areas; country of birth; alcohol intake; smoking status; physical activity; sex; Mediterranean diet score; sugar-sweetened soft drink consumption; and waist circumference. No adjustment for age and body mass index (the reason for moderate ROB).
Bias in selection of participants for the study	Low	Participant selection for the cohort was not related to the exposure or outcome.
Bias in classification of exposures	Moderate	Exposure assessment was performed using measurement by FFQs.
Bias due to departures from intended exposures	Low	At baseline, participants completed a 121-item FFQ, including separate questions about the number of times in the past year they had consumed regular (sugar-sweetened) or diet beverages. Follow-up less than 20 years.
Bias due to missing data	Moderate	Reasons for exclusion were documented and unlikely to differ across exposures threshold. The study authors did not conduct analyses that addressed loss to follow-up and thus judged a moderate risk of bias.
Bias in measurement of outcomes	Low	Incident cancer cases were ascertained from the Victorian Cancer Registry or the Australian Cancer Database as the earliest diagnosis of an invasive or metastatic primary cancer.
Bias in selection of the reported result	Low	Study results were fully reported, including baseline and covariates distribution and stratified analyses according to many confounding factors. Statistical methods reported in the methods section were used and presented in the results.
Overall bias	Moderate	Four domains were found at moderate ROB.
Hur (2021) [6]		

Bias due to confounding	Low	Model adjusted for age; energy intake; race; height; body mass index; menopausal status and menopausal hormone use; family history of colorectal cancer; smoking status; physical activity; regular use of aspirin; regular use of non-steroidal anti-inflammatory drugs; current use of multivitamins; intake of alcohol, red and processed meat, dietary fiber; total folate and total calcium; Alternative Healthy Eating Index-2010 score without sugar-sweetened beverages and alcohol; and lower endoscopy due to screening or for other indications within the past 10 years.
Bias in selection of participants for the study	Low	Participant selection for the cohort was not related to the exposure or outcome.
Bias in classification of exposures	Moderate	Exposure assessment was performed using measurement by FFQs.
Bias due to departures from intended exposures	Moderate	In 1991 and every 4 years thereafter, beverage intake was assessed via validated semi-quantitative FFQs. Follow-up more than 20 years.
Bias due to missing data	NI	No mention of missing data.
Bias in measurement of outcomes	Low	Medical records or pathology reports for CRC diagnoses reported on a biennial questionnaire or lethal CRC cases identified from the National Death Index, tumor registries, or death certificates.
Bias in selection of the reported result	Low	Study results were fully reported, including baseline and covariates distribution and stratified analyses according to many confounding factors. Statistical methods reported in the methods section were used and presented in the results.
Overall bias	Moderate	Two domains were found at moderate ROB.
Lim (2006) [7]		
Bias due to confounding	Moderate	Model adjusted for age at entry, sex, ethnicity, BMI, and history of diabetes. No adjustment for smoking and energy intake/physical activity (the reason for moderate ROB).

Bias in selection of participants for the study	Low	Participant selection for the cohort was not related to the exposure or outcome.
Bias in classification of exposures	Moderate	Exposure assessment was performed using measurement by FFQs.
Bias due to departures from intended exposures	Low	Dietary intakes were derived from the baseline 124-item FFQ. Follow-up less than 20 years.
Bias due to missing data	Moderate	Reasons for exclusion were documented and unlikely to differ across exposures threshold. The study authors did not conduct analyses that addressed loss to follow-up and thus judged a moderate risk of bias.
Bias in measurement of outcomes	Low	Histologically confirmed incident cancer cases were ascertained from the eight state cancer registries up to December 31, 2000.
Bias in selection of the reported result	Low	Study results were fully reported, including baseline and covariates distribution and stratified analyses according to many confounding factors. Statistical methods reported in the methods section were used and presented in the results.
Overall bias	Moderate	Three domains were found at moderate ROB.
McCullough (2014) [8]		
Bias due to confounding	Low	Model adjusted for age at baseline, gender, history of diabetes, BMI, smoking status, energy intake, and sugar-sweetened beverage intake.
Bias in selection of participants for the study	Low	Participant selection for the cohort was not related to the exposure or outcome.
Bias in classification of exposures	Moderate	Participants completed a set of questionnaires.
Bias due to departures from intended exposures	Low	Diet was assessed in 1999 and updated in 2003 with the use of a modified Willett FFQ. Follow-up less than 20 years.

Bias due to missing data	Low	Reasons for exclusion were documented and unlikely to differ across exposures threshold. Although some subjects were lost to follow-up and the missing data were not described by exposure status, the study authors conducted analyses that addressed loss to follow-up and are likely to have removed any risk of bias and thus judged a low risk of bias.
Bias in measurement of outcomes	Moderate	Of the 1196 incident cases of lymphoid neoplasms in this analysis, 941 cases were initially identified by self-report and subsequently verified by obtaining medical records or by linkage with state cancer registries.
Bias in selection of the reported result	Low	Study results were fully reported, including baseline and covariates distribution and stratified analyses according to many confounding factors. Statistical methods reported in the methods section were used and presented in the results.
Overall bias	Moderate	Two domains were found at moderate ROB.
Navarrete-Munoz (2016) [9]		
Bias due to confounding	Low	Model adjusted for center; sex; age at recruitment; educational level; physical activity; smoking status; alcohol consumption; juice, nectar, and soft drink consumption; energy intake; diabetes; and BMI.
Bias in selection of participants for the study	Low	Participant selection for the cohort was not related to the exposure or outcome.
Bias in classification of exposures	Moderate	Participants completed country-specific validated dietary questionnaires at baseline.
Bias due to departures from intended exposures	Low	The usual diet over the previous 12 months was assessed with the use of country-specific validated dietary questionnaires at baseline. Follow-up less than 20 years.
Bias due to missing data	Low	Reasons for exclusion were documented and unlikely to differ across exposures threshold. Although some subjects were lost to follow-up and the missing data were not described by exposure status, the study authors conducted analyses that

		addressed loss to follow-up and are likely to have removed any risk of bias and thus judged a low risk of bias.
Bias in measurement of outcomes	Low	Incident cancer and mortality data were obtained from regional or national population-based cancer and mortality registers except in Germany, Greece, and France, where they came from a combination of methods including active follow-up through study participants, next-of-kin information, the use of health insurance records, and cancer and pathology registries.
Bias in selection of the reported result	Low	Study results were fully reported, including baseline and covariates distribution and stratified analyses according to many confounding factors. Statistical methods reported in the methods section were used and presented in the results.
Overall bias	Moderate	One domain was found at moderate ROB.
Romanos-Nanclares (2021, NHS) [10]		
Bias due to confounding	Moderate	Model adjusted for age, SSB intake, race, age at menarche, age at menopause, postmenopausal hormone use, oral contraceptive use history, parity and age at first birth, breastfeeding history, family history of breast cancer, history of benign breast disease, height, cumulatively updated alcohol intake, cumulatively updated total caloric intake, physical activity, BMI at age 18 years, a modified Alternate Healthy Eating Index score (with SSBs and alcohol removed), socioeconomic status, and the change in weight since age 18. No adjustment for smoking status (the reason for moderate ROB).
Bias in selection of participants for the study	Low	Participant selection for the cohort was not related to the exposure or outcome.
Bias in classification of exposures	Moderate	Exposure assessment was performed using measurement by FFQs.

Bias due to departures from intended exposures	Moderate	Diet was assessed with a validated FFQ administered by the NHS in 1980, 1984, and 1986, and every 4 years thereafter, and in the NHSII in 1991 and every 4 years thereafter. Follow-up more than 20 years.
Bias due to missing data	Moderate	Reasons for exclusion were documented and unlikely to differ across exposures threshold. The study authors did not conduct analyses that addressed loss to follow-up and thus judged a moderate risk of bias.
Bias in measurement of outcomes	Moderate	Invasive breast cancer cases were identified through self-reports on the biennial questionnaires with medical confirmation or through the National Death Index.
Bias in selection of the reported result	Low	Study results were fully reported, including baseline and covariates distribution and stratified analyses according to many confounding factors. Statistical methods reported in the methods section were used and presented in the results.
Overall bias	Moderate	Five domains were found at moderate ROB.
Romanos-Nanclares (2021, NHSII) [10]		
Bias due to confounding	Moderate	Model adjusted for age, SSB intake, race, age at menarche, age at menopause, postmenopausal hormone use, oral contraceptive use history, parity and age at first birth, breastfeeding history, family history of breast cancer, history of benign breast disease, height, cumulatively updated alcohol intake, cumulatively updated total caloric intake, physical activity, BMI at age 18 years, a modified Alternate Healthy Eating Index score (with SSBs and alcohol removed), socioeconomic status, and the change in weight since age 18. No adjustment for smoking status (the reason for moderate ROB).

Bias in selection of participants for the study	Low	Participant selection for the cohort was not related to the exposure or outcome.
Bias in classification of exposures	Moderate	Exposure assessment was performed using measurement by FFQs.
Bias due to departures from intended exposures	Moderate	Diet was assessed with a validated FFQ administered by the NHS in 1980, 1984, and 1986, and every 4 years thereafter, and in the NHSII in 1991 and every 4 years thereafter. Follow-up more than 20 years.
Bias due to missing data	Moderate	Reasons for exclusion were documented and unlikely to differ across exposures threshold. The study authors did not conduct analyses that addressed loss to follow-up and thus judged a moderate risk of bias.
Bias in measurement of outcomes	Moderate	Invasive breast cancer cases were identified through self-reports on the biennial questionnaires with medical confirmation or through the National Death Index.
Bias in selection of the reported result	Low	Study results were fully reported, including baseline and covariates distribution and stratified analyses according to many confounding factors. Statistical methods reported in the methods section were used and presented in the results.
Overall bias	Moderate	Five domains were found at moderate ROB.
Schernhammer (2005, HPFUS) [11]		
Bias due to confounding	Low	Model adjusted for age, gender, follow-up cycle, history of diabetes, smoking status, quintiles of caloric intake, non-vigorous physical activity, and other soft drink consumption.
Bias in selection of participants for the study	Low	Participant selection for the cohort was not related to the exposure or outcome.

Bias in classification of exposures	Moderate	Exposure assessment was performed using measurement by FFQs.
Bias due to departures from intended exposures	Low	In 1986, the baseline questionnaire for the HPFS cohort included a 131-item semi-quantitative FFQ. The same five items of soft drink consumption as in the NHS were assessed, with an update in 1990, 1994, and 1998. Follow-up less than 20 years.
Bias due to missing data	NI	No mention of missing data.
Bias in measurement of outcomes	Moderate	With permission from study participants, pancreatic cancer was confirmed through physicians' review of medical records.
Bias in selection of the reported result	Low	Study results were fully reported, including baseline and covariates distribution and stratified analyses according to many confounding factors. Statistical methods reported in the methods section were used and presented in the results.
Overall bias	Moderate	Two domains were found at moderate ROB.
Schernhammer (2005, NHS) [11]		
Bias due to confounding	Low	Model adjusted for age, gender, follow-up cycle, history of diabetes, smoking status, quintiles of caloric intake, non-vigorous physical activity, and other soft drink consumption.
Bias in selection of participants for the study	Low	Participant selection for the cohort was not related to the exposure or outcome.
Bias in classification of exposures	Moderate	Exposure assessment was performed using measurement by FFQs.
Bias due to departures from intended exposures	Low	Diet was assessed with a validated FFQ administered by the NHS in 1980, 1984, and 1986, and every 4 years thereafter, and in the NHSII in 1991 and every 4 years thereafter. Follow-up less than 20 years.
Bias due to missing data	NI	No mention of missing data.

Bias in measurement of outcomes	Moderate	With permission from study participants, pancreatic cancer was confirmed through physicians' review of medical records.
Bias in selection of the reported result	Low	Study results were fully reported, including baseline and covariates distribution and stratified analyses according to many confounding factors. Statistical methods reported in the methods section were used and presented in the results.
Overall bias	Moderate	Two domains were found at moderate ROB.
Inoue-Choi (2013) [12]		
Bias due to confounding	Low	Model adjusted for age, smoking, physical activity, alcohol use, estrogen use, age at menarche, age at menopause, number of live births, coffee intake, and BMI.
Bias in selection of participants for the study	Low	Participant selection for the cohort was not related to the exposure or outcome.
Bias in classification of exposures	Moderate	Exposure assessment was performed using measurement by FFQs.
Bias due to departures from intended exposures	Low	Dietary intake was assessed using the Harvard Food Frequency Questionnaire (FFQ) at the study baseline. Follow-up less than 20 years.
Bias due to missing data	NI	No mention of missing data.
Bias in measurement of outcomes	Low	Incident cancers were identified via annual linkage with the State Health Registry of Iowa, part of the National Cancer Institute's Surveillance Epidemiology and End Results (SEER) program.
Bias in selection of the reported result	Low	Study results were fully reported, including baseline and covariates distribution and stratified analyses according to many confounding factors. Statistical methods reported in the methods section were used and presented in the results.
Overall bias	Moderate	One domain was found at moderate ROB.

Schernhammer (2012, HPFUS) [13]		
Bias due to confounding	Low	Model adjusted for age; questionnaire cycle; sugar-sweetened soda consumption; fruit and vegetable consumption; multivitamin use; intakes of alcohol, saturated fat, and animal protein; total energy; race; BMI; height; discretionary physical activity; smoking history; and menopausal status and use of hormone replacement therapy (women only).
Bias in selection of participants for the study	Low	Participant selection for the cohort was not related to the exposure or outcome.
Bias in classification of exposures	Moderate	Exposure assessment was performed using measurement by FFQs.
Bias due to departures from intended exposures	Low	In 1986, the baseline questionnaire for the HPFS cohort included a 131-item semi-quantitative FFQ. The same five items of soft drink consumption as in the NHS were assessed, with an update in 1990, 1994, and 1998. Follow-up less than 20 years.
Bias due to missing data	Moderate	Reasons for exclusion were documented and unlikely to differ across exposures threshold. The study authors did not conduct analyses that addressed loss to follow-up and thus judged a moderate risk of bias.
Bias in measurement of outcomes	Moderate	Participants were asked to report all incident cancer diagnoses and for each cancer report, permission to obtain medical records was confirmed.
Bias in selection of the reported result	Low	Study results were fully reported, including baseline and covariates distribution and stratified analyses according to many confounding factors. Statistical methods reported in the methods section were used and presented in the results.
Overall bias	Moderate	Three domains were found at moderate ROB.
Schernhammer (2012, NHS) [13]		

Bias due to confounding	Low	Model adjusted for age; questionnaire cycle; sugar-sweetened soda consumption; fruit and vegetable consumption; multivitamin use; intakes of alcohol, saturated fat, and animal protein; total energy; race; BMI; height; discretionary physical activity; smoking history; and menopausal status and use of hormone replacement therapy (women only).
Bias in selection of participants for the study	Low	Participant selection for the cohort was not related to the exposure or outcome.
Bias in classification of exposures	Moderate	Exposure assessment was performed using measurement by FFQs.
Bias due to departures from intended exposures	Low	Diet was assessed with a validated FFQ administered by the NHS in 1980, 1984, and 1986, and every 4 years thereafter, and in the NHSII in 1991 and every 4 years thereafter. Follow-up less than 20 years.
Bias due to missing data	Moderate	Reasons for exclusion were documented and unlikely to differ across exposures threshold. The study authors did not conduct analyses that addressed loss to follow-up and thus judged a moderate risk of bias.
Bias in measurement of outcomes	Moderate	Participants were asked to report all incident cancer diagnoses and for each cancer report, permission to obtain medical records was confirmed.
Bias in selection of the reported result	Low	Study results were fully reported, including baseline and covariates distribution and stratified analyses according to many confounding factors. Statistical methods reported in the methods section were used and presented in the results.
Overall bias	Moderate	Three domains were found at moderate ROB.
Zamora-Ros (2022) [14]		
Bias due to confounding	Low	Model adjusted for sex; center; age at recruitment; BMI; smoking status; physical activity; educational level; alcohol and energy intake; and for women: menopausal status, oral contraceptive use, and infertility problems.

Bias in selection of participants for the study	Low	Participant selection for the cohort was not related to the exposure or outcome.
Bias in classification of exposures	Moderate	Participants completed country-specific validated dietary questionnaires at baseline.
Bias due to departures from intended exposures	Low	The usual diet over the previous 12 months was assessed with the use of country-specific validated dietary questionnaires at baseline. Follow-up less than 20 years.
Bias due to missing data	Moderate	Reasons for exclusion were documented and unlikely to differ across exposures threshold. The study authors did not conduct analyses that addressed loss to follow-up and thus judged a moderate risk of bias.
Bias in measurement of outcomes	Low	Cancer incidence was determined through record linkage with national and regional cancer registries or via a combination of methods, including the use of health insurance records, contacts with cancer and pathology registries, and active follow-up evaluation of study participants and their next of kin.
Bias in selection of the reported result	Low	Study results were fully reported, including baseline and covariates distribution and stratified analyses according to many confounding factors. Statistical methods reported in the methods section were used and presented in the results.
Overall bias	Moderate	Two domains were found at moderate ROB.

Abbreviation: ROB, Risk of Bias; BMI, body mass index; ASB, artificially sweetened beverage; FFQ, Food Frequency Questionnaire.

Supplementary Table S3. Summary Risk of Bias (ROB) assessment with overall study-level risk of bias.*

Studies	Bias Due to Confounding	Bias in Selection of Participants for the Study	Bias in Measurement of Exposures	Bias due to Departures from Intended Exposures	Bias due to Missing Data	Bias in Measurement of Outcomes	Bias in Selection of the Reported Result	Overall Bias
Bao (2008) [1]	Low	Low	Moderate	Low	Low	Low	Low	Moderate
Bassett (2020) [2]	Moderate	Low	Moderate	Low	Moderate	Low	Low	Moderate
Chazelas (2019) [3]	Low	Low	Moderate	Low	NI	Moderate	Low	Moderate
Debras (2022) [4]	Low	Low	Moderate	Low	Moderate	Moderate	Low	Moderate
Hodge (2018) [5]	Moderate	Low	Moderate	Moderate	Moderate	Low	Low	Moderate
Hur (2021) [6]	Low	Low	Moderate	Low	NI	Low	Low	Moderate
Lim (2006) [7]	Moderate	Low	Moderate	Low	Moderate	Low	Low	Moderate
McCullough (2014) [8]	Low	Low	Moderate	Low	Low	Moderate	Low	Moderate
Navarrete-Munoz (2016) [9]	Low	Low	Moderate	Low	Low	Low	Low	Moderate
Romanos-Nanclares (2021, NHS) [10]	Moderate	Low	Moderate	Moderate	Moderate	Moderate	Low	Moderate
Romanos-Nanclares (2021, NHSII) [10]	Moderate	Low	Moderate	Moderate	Moderate	Moderate	Low	Moderate
Schernhammer (2005, HPFUS) [11]	Low	Low	Moderate	Low	NI	Moderate	Low	Moderate
Schernhammer (2005, NHS) [11]	Low	Low	Moderate	Low	NI	Moderate	Low	Moderate
Inoue-Choi (2013) [12]	Low	Low	Moderate	Low	NI	Low	Low	Moderate
Schernhammer (2012, HPFUS) [13]	Low	Low	Moderate	Low	Moderate	Moderate	Low	Moderate
Schernhammer (2012, NHS) [13]	Low	Low	Moderate	Low	Moderate	Moderate	Low	Moderate
Zamora Ros (2022) [14]	Low	Low	Moderate	Low	Moderate	Low	Low	Moderate

*: Green for low risk of bias and yellow for moderate risk of bias.

Supplementary Table S4. Minimally and maximally adjusted RR by cancer type.

Cancer Type	Minimal Adjustment						Maximal adjustment[6]					
	n	***RR*** **(95% CI)**	*p*	I^2 **(%)**	*p-Heterogeneity*	**tau**^{2}	*n*	***RR*** **(95% CI)**	*p*	I^2 **(%)**	*p-Heterogeneity*	**tau**^{2}
Hodgkin lymphoma	0	.	.	.	.	.	1	0.77(0.45–1.33)	0.351	.	.	.
Leukemia	2	1.43(1.02–1.99)[1]	0.038	0.0%	0.773	0.000	3	1.35(1.03–1.77)	0.030	0.0%	0.880	0.000
Multiple myeloma	2	1.27(0.60–2.71)[1]	0.535	77.6%	0.035	0.231	3	1.18(0.69–2.02)	0.537	68.9%	0.040	0.153
Non-Hodgkin lymphoma	2	1.12(0.85–1.48)[1]	0.418	62.6%	0.102	0.025	4	1.05(0.91–1.21)	0.506	16.3%	0.310	0.004
Colorectal cancer	1	0.86(0.50–1.47)[2]	0.581	.	.	.	3	0.78(0.62–0.99)	0.037	0.0%	0.966	0.000
Gastric cancer	0	.	.	.	.	.	1	1.03(0.53–1.99)	0.930	.	.	.
Pancreatic cancer	4	1.11(0.94–1.31)[3]	0.230	0.0%	0.823	0.000	4	1.10(0.92–1.31)	0.307	0.0%	0.560	0.000
Breast cancer	3	1.00(0.87–1.14)[4]	0.963	80.5%	0.006	0.011	4	0.99(0.90–1.08)	0.746	50.3%	0.110	0.004
Endometrial cancer	0	.	.	.	.	.	2	0.81(0.64–1.03)	0.091	0.0%	0.997	0.000
Ovarian cancer	0	.	.				1	1.37(0.72–2.61)	0.338	.	.	.
Prostate cancer	1	1.20(0.90–1.60)[4]	0.209	.	.	.	2	1.06(0.70–1.62)	0.785	55.5%	0.134	0.054
Gliomas	0	.	.	.	.	.	1	0.73(0.46–1.15)	0.178	.	.	.
Kidney cancer	0	.	.	.	.	.	1	1.02(0.96–1.08)	0.814	.	.	.
Thyroid cancer	1	1.26(0.87–1.83)[5]	0.223	.	.	.	1	1.16(0.80–1.69)	0.437	.	.	.
Overall cancer	11	1.08(0.97–1.20)	0.297	70.6%	<0.001	0.016	17	1.03(0.96–1.11)	0.407	53.0%	0.005	0.009

[1] Adjusted for age and questionnaire cycle only. [2] Adjusted for age and energy only. [3] Two adjusted for age only. One adjusted for age and sex. One adjusted for age, sex, and center. [4] Adjusted for age only. [5] Adjusted for age, sex, and center. [6] Most studies adjusted for sociodemographic factors, lifestyle factors, and adiposity measures. Detailed information about confounders of each study is presented in Table 1.

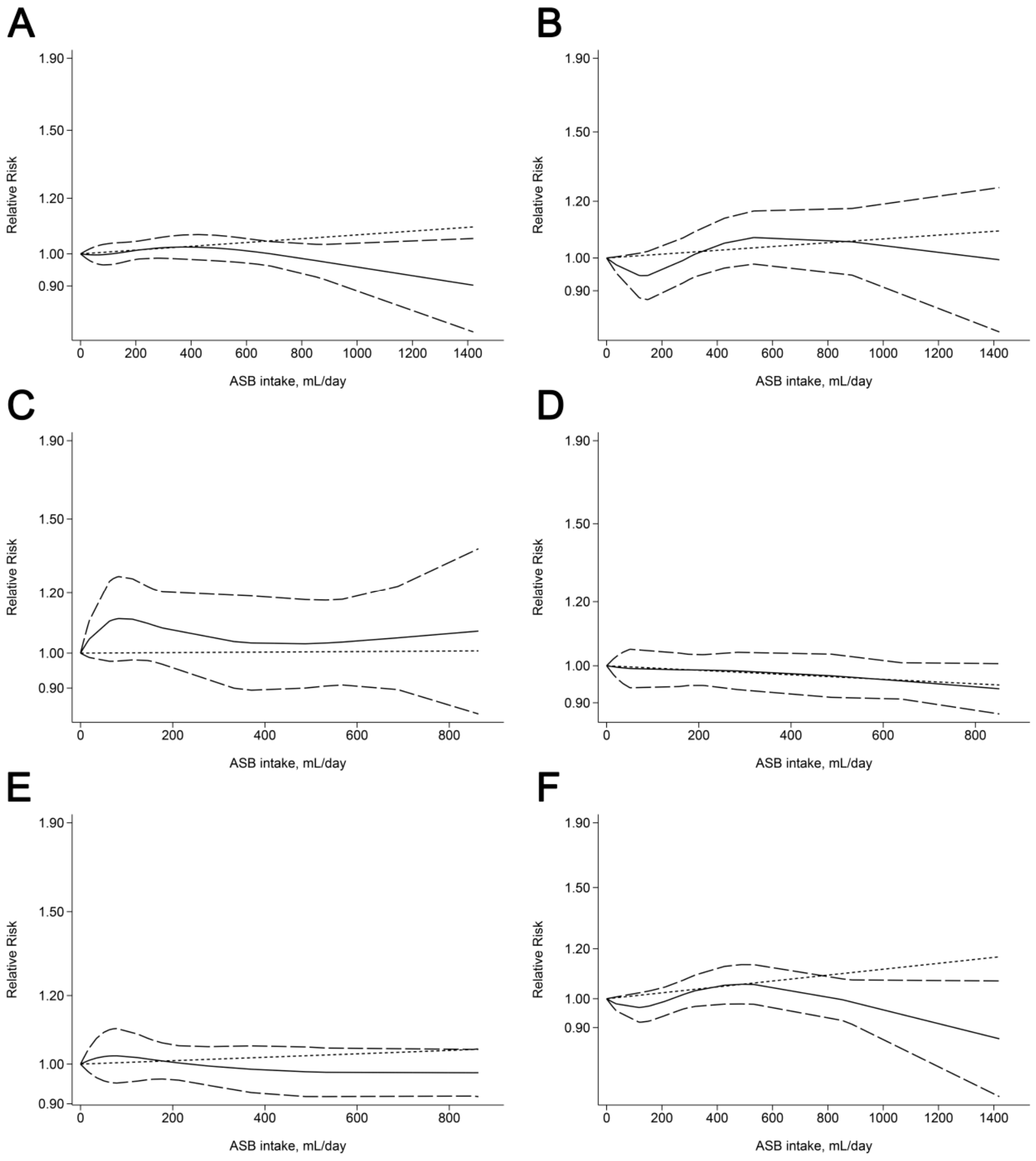

Supplementary Figure S1. Dose-response analysis of artificially sweetened beverage (ASB) intake with risk of cancers. The shot dash dot line represents the linear model RR. The solid line and the long dash line represent the RR estimates and 95% CI of the non-linear model, respectively. (**A**) Association between ASB intake and overall cancer risk. (**B**) Association between ASB intake and risk of hematopoietic cancer. (**C**) Association between ASB intake and risk of digestive system cancer. (**D**) Association between ASB intake and risk of female hormone-related cancer. (**E**) Association between ASB intake and risk of obesity-related cancer. (**F**) Association between ASB intake and risk of non-

obesity-related cancer.

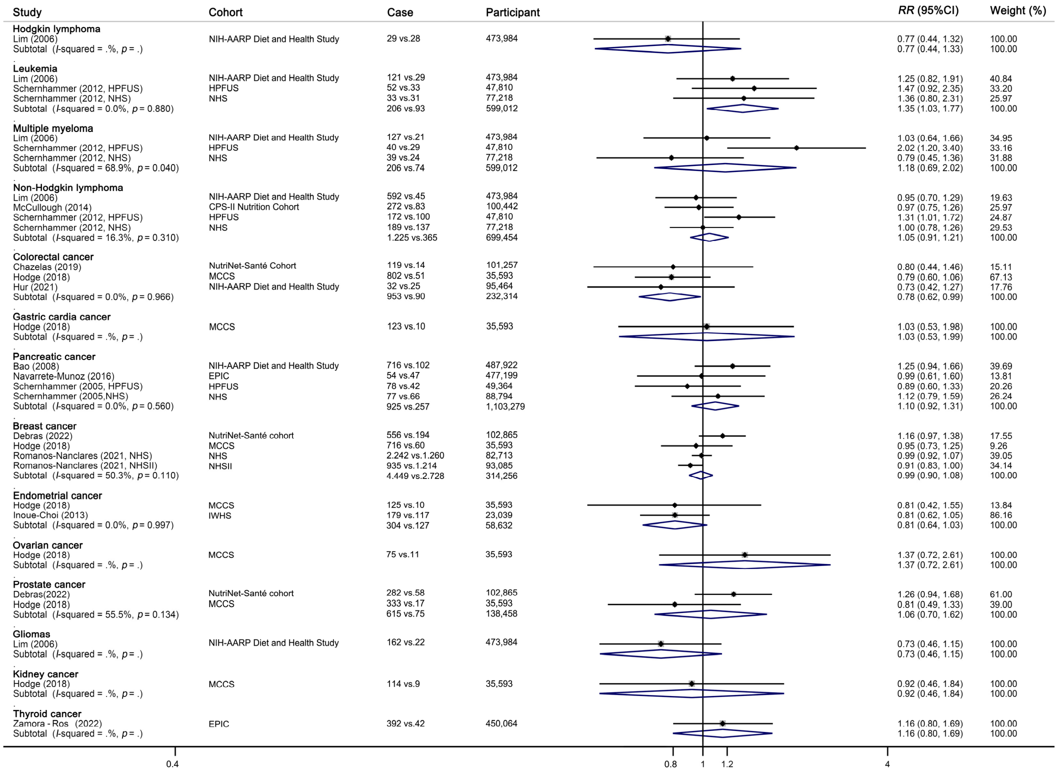

Supplementary Figure S2. Forest plot of artificially sweetened beverage (ASB) intake and risk of site-specific cancers. Dots indicate adjusted RRs by comparing the highest with the lowest categories of intake, and diamonds indicate the pooled RRs. The size of the shaded square is proportional to the percentage weight of each study, and horizontal lines indicate 95% CIs. Overall RRs calculated with a random-effect model. Cases of cancer in lowest versus highest categories and participants for each study are presented. Abbreviation: NIH-AARP Diet and Health Study, the National Institutes of Health–AARP Diet and Health Study; HPFUS, the Health Professionals Follow-Up Study; NHS, the Nurses' Health Study; CPS-II Nutrition Cohort, the Cancer Prevention Study-II Nutrition Cohort; MCCS, the Melbourne Collaborative Cohort Study; EPIC, European Prospective Investigation into Cancer and Nutrition; IWHS, the Iowa Women's Health Study; NHS II, and the Nurses' Health Study II.

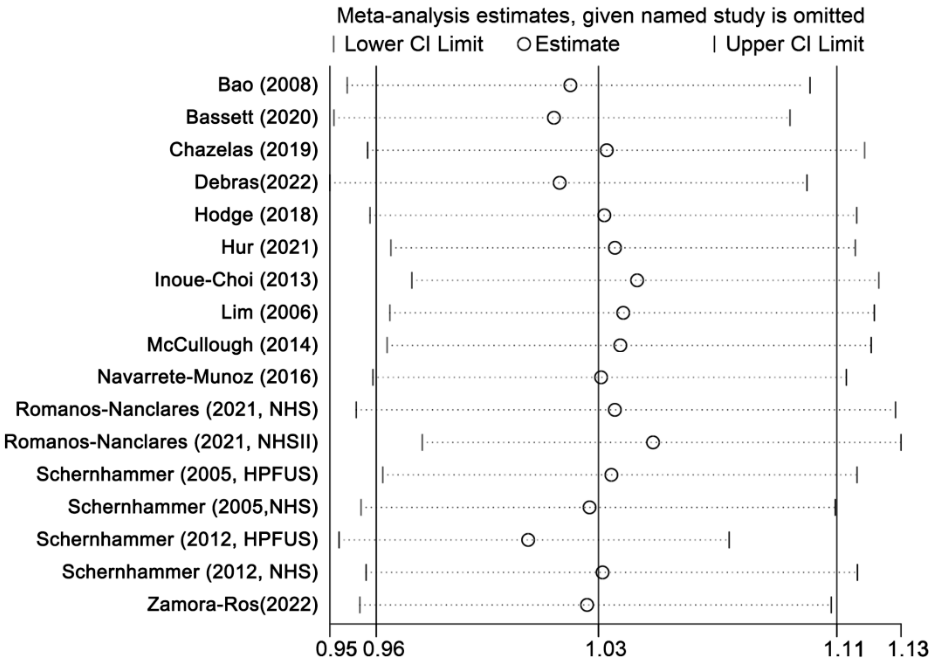

Supplementary Figure S3. Sensitivity analysis of studies included in the meta-analysis on ASB intake and overall cancer risk [1-14].

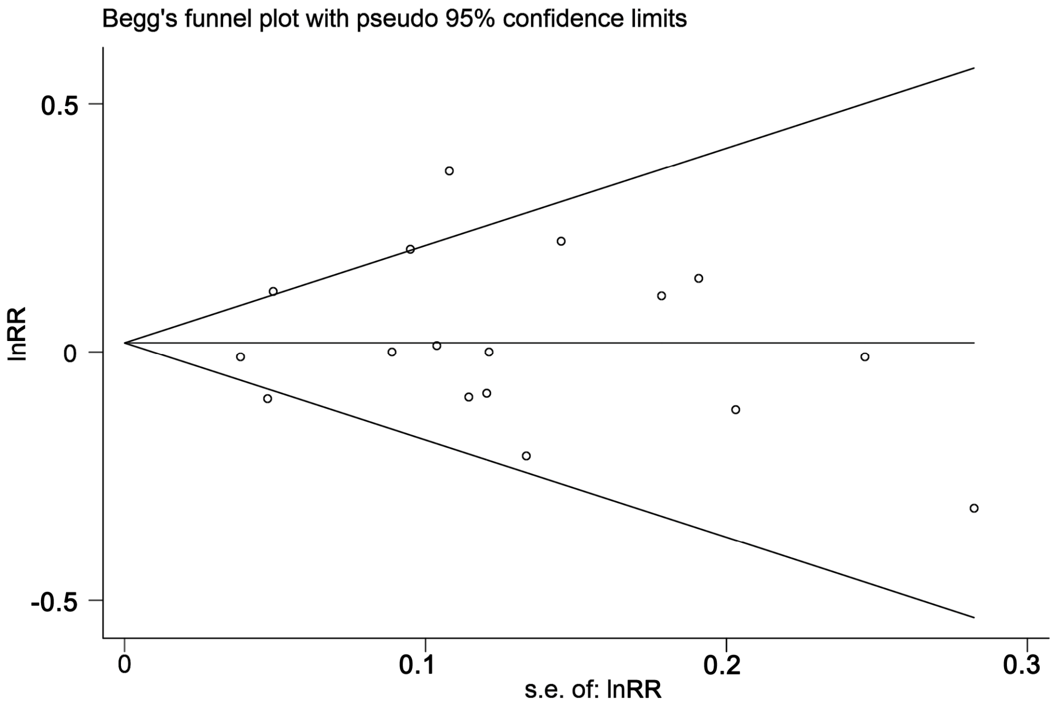

Supplementary Figure S4. Funnel plots of studies included in the meta-analysis. Each circle represented an included cohort. The horizontal line in the middle represents the combined RR of included 17 cohorts. The two slashes represent the 95%CI of combined RR.

Reference:

1. Bao, Y.; Stolzenberg-Solomon, R.; Jiao, L.; Silverman, D.T.; Subar, A.F.; Park, Y.; Leitzmann, M.F.; Hollenbeck, A.; Schatzkin, A.; Michaud, D.S. Added sugar and sugar-sweetened foods and beverages and the risk of pancreatic cancer in the National Institutes of Health-AARP Diet and Health Study. *Am J Clin Nutr* **2008**, 88, 431-40.
2. Bassett, J.K.; Milne, R.L.; English, D.R.; Giles, G.G.; Hodge, A.M. Consumption of sugar-sweetened and artificially sweetened soft drinks and risk of cancers not related to obesity. *Int. J. Cancer* **2020**, *146*, 3329–3334.
3. Chazelas, E.; Srour, B.; Desmetz, E.; Kesse-Guyot, E.; Julia, C.; Deschamps, V.; Druesne-Pecollo, N.; Galan, P.; Hercberg, S.; Latino-Martel, P.; et al. Sugary drink consumption and risk of cancer: results from NutriNet-Santé prospective cohort. *Bmj* **2019**, 366, l2408.
4. Debras, C.; Chazelas, E.; Srour, B.; Druesne-Pecollo, N.; Esseddik, Y.; Szabo de Edelenyi, F.; Agaësse, C.; de Sa, A.; Lutchia, R.; Gigandet, S.; et al. Artificial sweeteners and cancer risk: Results from the NutriNet-Santé population-based cohort study. *PLoS Med.* **2022**, *19*, e1003950.
5. Hodge, A.M.; Bassett, J.K.; Milne, R.L.; English, D.R.; Giles, G.G. Consumption of sugar-sweetened and artificially sweetened soft drinks and risk of obesity-related cancers. *Public Health Nutr.* **2018**, *21*, 1618–1626.
6. Hur, J.; Otegbeye, E.; Joh, H.K.; Nimptsch, K.; Ng, K.; Ogino, S.; Meyerhardt, J.A.; Chan, A.T.; Willett, W.C.; Wu, K.; et al. Sugar-sweetened beverage intake in adulthood and adolescence and risk of early-onset colorectal cancer among women. *Gut* **2021**, *70*, 2330–2336.
7. Lim, U.; Subar, A.F.; Mouw, T.; Hartge, P.; Morton, L.M.; Stolzenberg-Solomon, R.; Campbell, D.; Hollenbeck, A.R.; Schatzkin, A. Consumption of aspartame-containing beverages and incidence of hematopoietic and brain malignancies. *Cancer Epidemiol. Biomarkers Prev.* **2006**, *15*, 1654–1659.
8. McCullough, M.L.; Teras, L.R.; Shah, R.; Diver, W.R.; Gaudet, M.M.; Gapstur, S.M. Artificially and sugar-sweetened carbonated beverage consumption is not associated with risk of lymphoid neoplasms in older men and women. *J. Nutr.* **2014**, *144*, 2041–2049.
9. Navarrete-Muñoz, E.M.; Wark, P.A.; Romaguera, D.; Bhoo-Pathy, N.; Michaud, D.; Molina-Montes, E.; Tjønneland, A.; Olsen, A.; Overvad, K. Sweet-beverage consumption and risk of pancreatic cancer in the European Prospective Investigation into Cancer and Nutrition (EPIC). *Am. J. Clin. Nutr.* **2016**, *104*, 760–768.
10. Romanos-Nanclares, A.; Collins, L.C.; Hu, F.B.; Willett, W.C.; A Rosner, B.; Toledo, E.; Eliassen, A.H. Sugar-Sweetened Beverages, Artificially Sweetened Beverages, and Breast Cancer Risk: Results From 2 Prospective US Cohorts. *J. Nutr.* **2021**, *151*, 2768–2779.
11. Schernhammer, E.S.; Hu, F.B.; Giovannucci, E.; Michaud, D.S.; Colditz, G.A.; Stampfer, M.J.; Fuchs, C.S. Sugar-sweetened soft drink consumption and risk of pancreatic cancer in two prospective cohorts. *Cancer Epidemiol Biomarkers Prev* **2005**, 14, 2098-105.
12. Inoue-Choi, M.; Robien, K.; Mariani, A.; Cerhan, J.R.; Anderson, K.E. Sugar-sweetened beverage intake and the risk of type I and type II endometrial cancer among postmenopausal women. *Cancer Epidemiol Biomarkers Prev* **2013**, 22, 2384-94.
13. Schernhammer, E.S.; Bertrand, K.A.; Birmann, B.M.; Sampson, L.; Willett, W.C.; Feskanich, D. Consumption of artificial sweetener- and sugar-containing soda and risk of lymphoma and leukemia in men and women. *Am. J. Clin. Nutr.* **2012**, *96*, 1419–1428.
14. Zamora-Ros, R.; Cayssials, V.; Clèries, R.; Torrents, M.; Byrnes, G.; Weiderpass, E.; Sandström, M.; Almquist, M.; Boutron-Ruault, M.-C.; Tjønneland, A.; et al. Sweetened beverages are associated with a higher risk of differentiated thyroid cancer in the EPIC cohort: A dietary pattern approach. *Eur. J. Nutr.* **2022**. https://doi.org/10.1007/s00394-022-02953-5.
